# A clinically relevant heterozygous ATR mutation sensitizes colorectal cancer cells to replication stress

**DOI:** 10.1038/s41598-022-09308-4

**Published:** 2022-03-31

**Authors:** Tom Egger, Benoît Bordignon, Arnaud Coquelle

**Affiliations:** 1 IRCM, Institut de Recherche en Cancérologie de Montpellier, INSERM U1194, Université de Montpellier, Institut Régional du Cancer de Montpellier, 34298 Montpellier, France; 2 Institut de Génétique Humaine, UMR9002, CNRS-Université de Montpellier, 34396 Montpellier, France; 3 Montpellier Ressources Imagerie, BioCampus, University of Montpellier, CNRS, INSERM, Montpellier, France

**Keywords:** DNA replication, Cancer genetics, Chemotherapy

## Abstract

Colorectal cancer (CRC) ranks third among the most frequent malignancies and represents the second most common cause of cancer-related deaths worldwide. By interfering with the DNA replication process of cancer cells, several chemotherapeutic molecules used in CRC therapy induce replication stress (RS). At the cellular level, this stress is managed by the ATR-CHK1 pathway, which activates the replication checkpoint. In recent years, the therapeutic value of targeting this pathway has been demonstrated. Moreover, MSI + (microsatellite instability) tumors frequently harbor a nonsense, heterozygous mutation in the ATR gene. Using isogenic HCT116 clones, we showed that this mutation of ATR sensitizes the cells to several drugs, including SN-38 (topoisomerase I inhibitor) and VE-822 (ATR inhibitor) and exacerbates their synergistic effects. We showed that this mutation bottlenecks the replication checkpoint leading to extensive DNA damage. The combination of VE-822 and SN-38 induces an exhaustion of RPA and a subsequent replication catastrophe. Surviving cells complete replication and accumulate in G2 in a DNA-PK-dependent manner, protecting them from cell death. Together, our results suggest that RPA and DNA-PK represent promising therapeutic targets to optimize the inhibition of the ATR-CHK1 pathway in oncology. Ultimately, ATR frameshift mutations found in patients may also represent important prognostic factors.

## Introduction

Colorectal cancer (CRC) is the second most common cause of cancer related deaths worldwide. To date, chemotherapy remains one of the cornerstones of CRC treatments, *FOLFOX*, *FOLFIRI* and variants being amongst the most commonly prescribed therapeutic regimens^1,2^. By impairing DNA replication, these drugs essentially induce replication stress (RS)^3–5^. ATR (Ataxia Telangiectasia and Rad3-related) is the main sensor of RS^6–9^. Replication protein A (RPA) -coated single stranded DNA arising from the impairment of replication fork progression promotes the recruitment of the ATRIP-ATR complex^10^. TopBP1^11,12^ and ETAA1^13^ stimulate the kinase activity of ATR, leading to the efficient phosphorylation of downstream targets, such as CHK1. Activation of CHK1 leads to various cellular processes including inhibition of new origin firing, delaying of cell cycle progression and recruitment of DNA repair factors^14–16^. Because this replication checkpoint is crucial for cancer cells displaying high RS, targeting the ATR-CHK1 axis in cancer therapy became a promising rationale^17^. Indeed, ATR and CHK1 inhibitors efficiently potentiate effects of DNA damaging agents in cancer cells^18,19^. SN-38 (active metabolite of irinotecan) stabilizes Topoisomerase I cleavage complexes (Top1CCs) onto DNA, which represent obstacles to replication fork progression^20–22^. Collisions between replication forks and Top1CC are known to induce single-ended double strand breaks, highly cytotoxic structures that are managed by the Break-Induced Replication (BIR) pathway^23^. The ssDNA intermediates generated during these Homologous Recombination (HR) processes is stabilized by RPA. This RPA protection is known to be rate limiting during the cellular response to replication inhibitors such as hydroxyurea and aphidicolin^24,25^. RPA exhaustion leads to replication catastrophe involving irreversible DNA damage, a particularly detrimental outcome for cells. ATR is known to protect cells from the exhaustion of RPA via its regulation of origin firing^24^. However, it is not known if RPA is also a rate limiting factor in contexts that don’t involve uncoupling events, such as the management of Top1CCs which, by a matter of size, do not allow replicative helicases to go through^26^.

Moreover, around 40% of CRCs with microsatellite instability (MSI +) harbor a heterozygous frameshift mutation in the ATR gene (exon 10), creating a premature stop codon within ATR’s N-HEAT domain^27,28^. Therefore, MSI + tumors harboring this mutation only rely on a single functional ATR allele. Since the ATR pathway holds a major role in the replication stress response, we hypothesized that this mutation could impact the cellular response to RS. To test this hypothesis, we designed an isogenic cellular model in HCT116 (CRC, MSI + cancer cell line), which showed that ATR mutation strongly sensitizes cells to SN-38 and VE-822 (ATR inhibitor), both alone and combined. Our results show that the ATR mutation dramatically increases the caspase-3 dependent apoptosis and DNA damage induced by SN-38 ± VE-822. We show that the inhibition of ATR by VE-822 allows cells to bypass the early-S cell cycle arrest induced by SN-38. Our analyses demonstrate this failure of the replication checkpoint leads to an exhaustion of RPA and a subsequent replication catastrophe, with the ATR mutation predisposing cells to these detrimental outcomes. Cells surviving to the SN-38 + VE-822 combination complete the replication and accumulate in G2 in a DNA-PK-dependent manner. This post-replicative checkpoint protects cells from mitotic catastrophe, most likely via the phosphorylation of RPA. Together, our results suggest that RPA and DNA-PK represent promising therapeutic targets to achieve synthetic lethal interactions with DNA damaging agents and ATR inhibitors. Besides, our study represents a proof of concept to use the ATR mutational status as predictive chemosensitivity biomarker to topoisomerase I and ATR inhibitors in cancer therapy.

## Results

### A relevant cellular model to study the heterozygous frameshift mutation of ATR found in MSI + tumors

**Figure 1:**
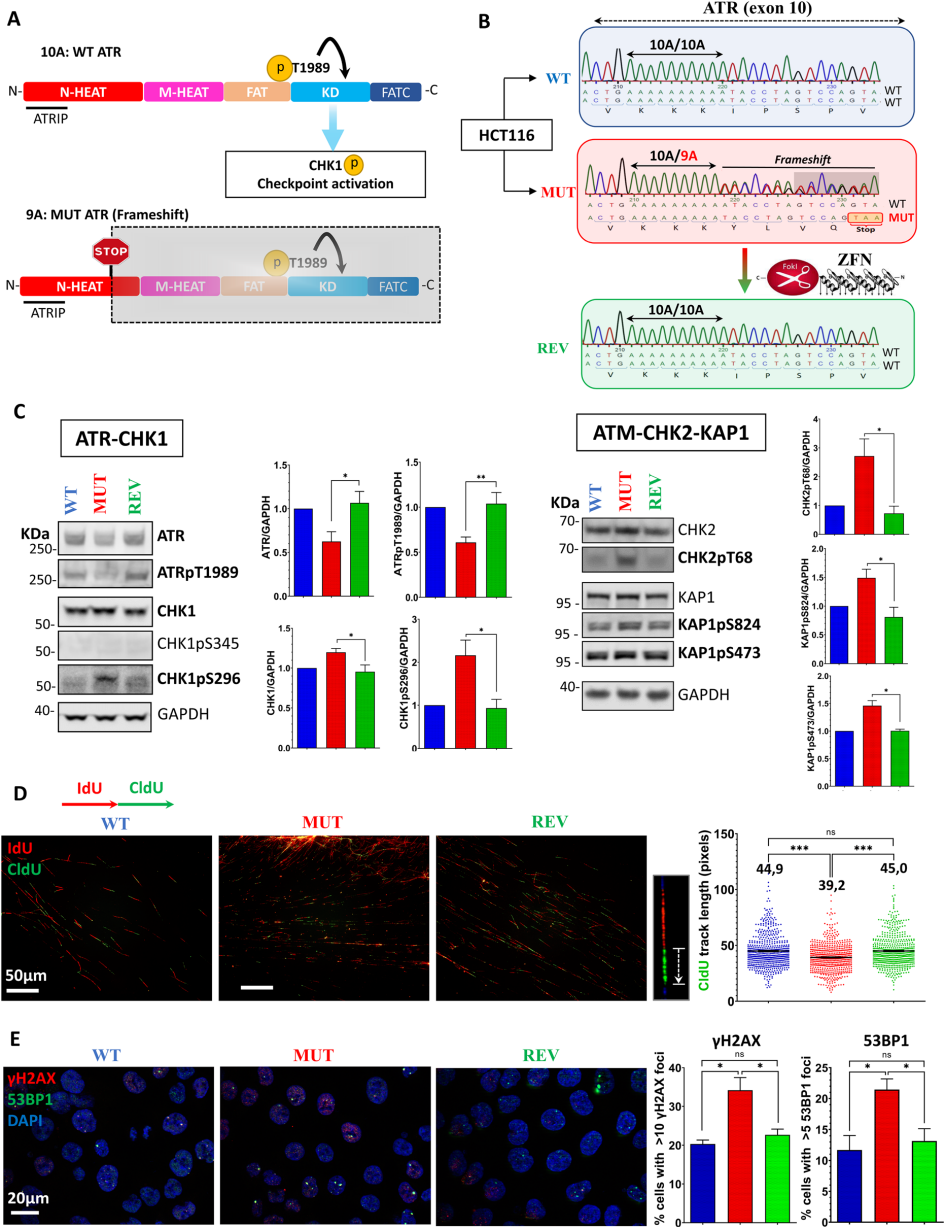
The clinically relevant ATR mutation is associated with a dysregulation of the ATR/M activities, endogenous replication stress and DNA damage. (**A**) Schematic representation of the ATR peptide with its functional domains. The “9A” frameshift mutation induces a stop codon in ATR’s ORF (Stop sign). Hence, this mutant allele is unable to code for the catalytic C-terminal domains of ATR. (**B**) Wild-type (WT, blue) and mutant (MUT, red) ATR clones were isolated from HCT116 by limit dilution. The poly-A region located in ATR’s exon 10 was sequenced. WT harbors the homozygous wild-type ATR sequence, whereas MUT harbors the clinically significant heterozygous frameshift mutation, leading to a downstream premature stop codon (red box). A revertant “REV” (green) clone has been obtained by genetic engineering of MUT using ZFN nucleases to restore a homozygous wild-type ATR genotype while conserving the genetic background of MUT. (**C)** Western Blot analysis of the ATR-CHK1 and ATM-CHK2-KAP1 pathways.GAPDH served as a loading control for each individual blot (see [Fig. S5]). A representative GAPDH panel is shown. The quantifications (relative to WT) were calculated from 3 to 5 biological replicates. Mann–Whitney tests were applied. (Full blots: [Fig. S5]) (**D**) DNA fiber assay performed in ATR mutant cells. IdU and CldU were sequentially added to culture media. DNA was manually spread, and fibers were observed (× 400). Representative images are shown. IdU-positive CldU tracks were measured using ImageJ (exclusion of origin firing and termination events). (*n* = 600, 200 fibers × 3 independent experiments). Means are displayed on the graphs. Mann–Whitney significance tests were applied. (**E**) *γ*-H2AX (red) and 53BP1 (green) immunofluorescent detection. DNA was counterstained with DAPI (blue). Cells were observed with a Zeiss Axio Imager with Apotome (× 63 objective). 53BP1 and *γ*H2AX foci were scored in > 200 cells per condition. The experiment was repeated 3 times (total > 600 cells) and the percentage of cells harboring > 10 γH2AX or > 5 53BP1 foci were plotted on graphs. Mann–Whitney significance tests were applied.

We designed a cellular model to study the clinically relevant ATR heterozygous mutation found in MSI + cancers. This mutation leads to the appearance of a premature stop codon on the “9A” mutant allele (N-HEAT domain). Because most of the functional domains of ATR are located downstream, the mutant allele cannot produce a functional ATR peptide [Fig. 1A]. We used the well described MSI + CRC cell line HCT116 to study the putative impacts of this genetic alteration. To do so, we isolated spontaneous wild-type (WT) and mutant (MUT) ATR HCT116 cells and raised clones from them. ATR genotyping was performed following a PCR amplification of the mutated poly-adenine region (poly-A10, exon 10). The DNA sequences of the amplicons are shown in [Fig. 1B]. “WT” harbors the wild-type sequence of the ATR gene at this locus (“10A/10A”), whereas “MUT” harbors a heterozygous deletion of an adenine (A) within the poly-A microsatellite generating a premature stop codon (red box). As these two naturally occurring clones were isolated from the heterogeneous HCT116 cell line, none can exclude slight, non specific genomic variations. To rule out this potential bias, a ZFN driven genetic engineering allowed us to design an ATR « Revertant » clone from the mutant clone. Hence, this so called “REV” clone shares the same genetic background as MUT, while harboring the wild-type ATR sequence on both alleles, making it a powerful isogenic control.

We first checked the basal activities of the ATR-CHK1 and ATM-CHK2-KAP1 pathways in ATR mutant cells by western blotting [Fig. 1C]. We observed significant alterations in the expression and phosphorylation of ATR and CHK1 in MUT cells as compared with their isogenic counterpart REV. Interestingly, while ATR was less expressed and less phosphorylated at Thr1989, CHK1 was found upregulated and hyperphosphorylated at Ser296, but not Ser345. Persistent replication stress has been shown to induce this autophosphorylation response of CHK1 on Ser296, triggering of G1-S and G2-M checkpoints by targeting CDC25A via 14-3-3 for proteasomal degradation^29–31^. Consistent with this, the detection of total CDC25A is dramatically decreased in mutant cells when assessed by western blotting [Fig. S1A]. Interestingly, MUT cells showed an increased phosphorylation of CHK2 (Thr68) and KAP1 (Ser473 and 824), downstream targets of the ATM pathway. Altogether, these observations suggest that these alterations of the ATR-CHK1 pathway increase the level of endogenous RS. Besides, the increased ATM-CHK2-KAP1 signaling could potentially emerge from the generation of double strand breaks (DSBs), as this is a common outcome of RS.

To verify these assumptions, we studied replication fork progression (as a marker of replication stress) by DNA fiber assay [Fig. 1D] along with the detection of DSBs with the commonly-used ɣH2AX/53BP1 immunofluorescence co-staining [Fig. 1E]. We observed a significant impairment of replication fork progression in ATR mutant cells, with the track length phenotype being fully recovered in REV cells, suggesting that increased levels of endogenous replication stress are indeed associated with this ATR mutation [Fig. 1D]. These results were obtained by focusing on replication forks that were active at the beginning of the CldU pulse (i.e. IdU tracks followed by CldU tracks), ruling out the potential bias induced by replication forks that could fire during the pulses. Regarding the DSBs, immunofluorescent foci counting of ɣH2AX and 53BP1 showed increased values in MUT cells [Fig. 1E]. Western blot analyses of H2AX phosphorylation corroborated this experiment [Fig. S1B]. Interestingly, no difference in cell cycle distribution could be attributed to the ATR status of cells by BrdU/PI flow cytometry analysis [Fig. S1C]. Complementary pulse-chase experiments also showed that the ATR mutation has no impact on replication entry or progression [Fig. S1D].

Altogether, these results demonstrate that the ATR mutation correlates with altered ATR/M pathway activities, endogenous replication stress and increased endogenous DNA damage levels (notably DSBs) but no change in the cell cycle profile.

### The ATR heterozygous mutation found in MSI + tumors sensitizes cells to topoisomerase I and ATR inhibitors

**Figure 2:**
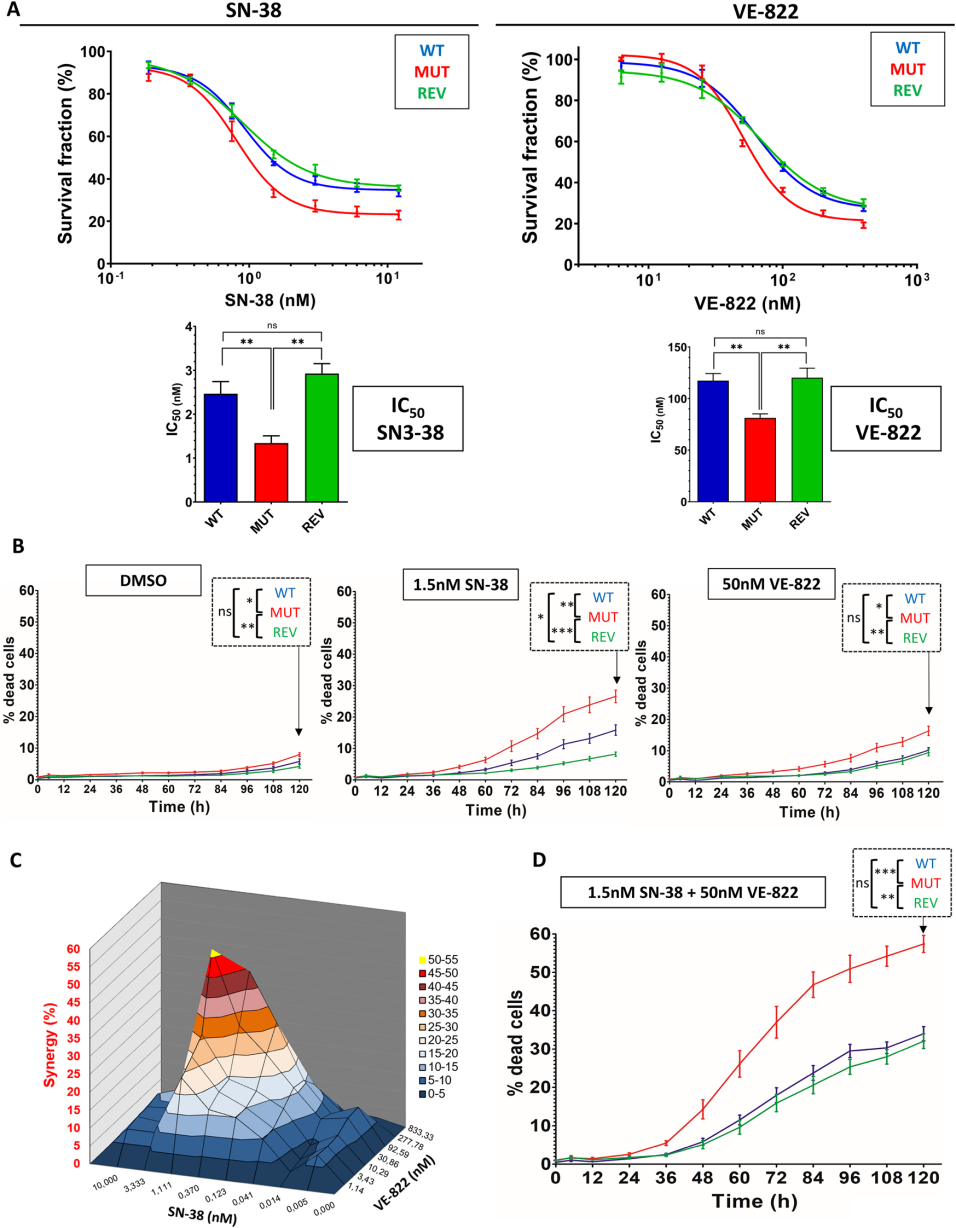
The ATR mutation sensitizes cells to SN-38 and VE-822, alone and combined. (**A**) Cells were treated with the indicated doses of SN-38 or VE-822 for 72 h. Sulforhodamine B assays were performed. Survival curves were plotted and IC_50_ (lower graphs) were calculated from 5 independent replicates. Mann–Whitney tests were applied. (**B**) Cells were treated with 1.5 nM SN-38 or 50 nM VE-822 and scanned every 12 h using the Celigo imager. Dead cells percentages were obtained with the CellTox Green Kit (Promega). Error bars represent s.e.m from 5 independent experiments. Mann–Whitney tests were applied on the last timepoint. (**C**) Synergy plot for an 8-doses combination of SN-38 and VE-822 on WT HCT116 cells. Plot was obtained using Mac Synergy on excel, doses of each drugs are displayed in (X) and (Y). Synergy (%) is displayed in (Z). The experiment was repeated 3 times. (**D**) Cells were treated with 1.5 nM SN-38 + 50 nM VE-822 and scanned on the Celigo imager. Error bars represent s.e.m from 5 independent replicates and Mann–Whitney tests were applied on the last time points.

Since it is known that the ATR-CHK1 replication checkpoint has a major role in drug response, we checked if this endogenous ATR deficiency has any impact on cell survival upon a panel of drugs. To do so, sulforhodamine survival assays (SRB) were performed. We showed that the ATR mutation increases cell sensitivity to several molecules. Amongst them, SN-38 (active metabolite of Irinotecan, topoisomerase I inhibitor) and VE-822 (ATR inhibitor) were those that showed the most dramatic sensitization of ATR mutant cells [Fig. 2A]. A twofold decrease of the IC_50_ of both SN-38 and VE-822 was observed in ATR mutant as compared with WT and REV cells. To discriminate between cytotoxic and cytostatic effects, we completed our analysis of SN-38 and VE-822 toxicity using Celigo Imaging Cytometer (Nexcelom Bioscience) on living cells. The percentage of dead cells was assessed using the CellTox kit (Promega), at doses of SN-38 and VE-822 matching their respective IC_50_ [Fig. 2B]. A significant increase in cell death was highlighted in ATR mutant cells for both molecules, although mutant cells also presented a slightly higher spontaneous death rate. As SN-38 is known to induce replication stress and notably ATR dependent responses, we combined both molecules in 72 h survival assays. The combination of both molecules resulted in highly synergistic effects, reaching a maximum of 50% [Fig. 2C] in WT cells. The maximum synergy was achieved at doses roughly matching the IC_50_ of each molecule in single drug treatments. The cytotoxic effects of the SN-38 + VE-822 combination was strongly enhanced in all clones as compared to single drug treatments, reflecting the synergistic effects observed with SRB assays. The ATR mutation was here linked to a twofold increase in cell death [Fig. 2D]. Similar but slightly weaker synergistic effects were highlighted between VE-822 and other widely-used replication stress inducers such as hydroxyurea (HU) and aphidicolin (APH), but not with the microtubule depolymerization inhibitor taxol, used here as a negative control since it does not target replicating cells [Fig. S2A]. Altogether, these results highlight the cytotoxic sensitization of ATR mutant cells to topoisomerase I and ATR inhibitors, either used separately or combined.

### The SN-38 + VE-822 combination triggers a caspase-3 dependent apoptosis as cells undergo extensive DNA damage due to a failure of the replication checkpoint

**Figure 3:**
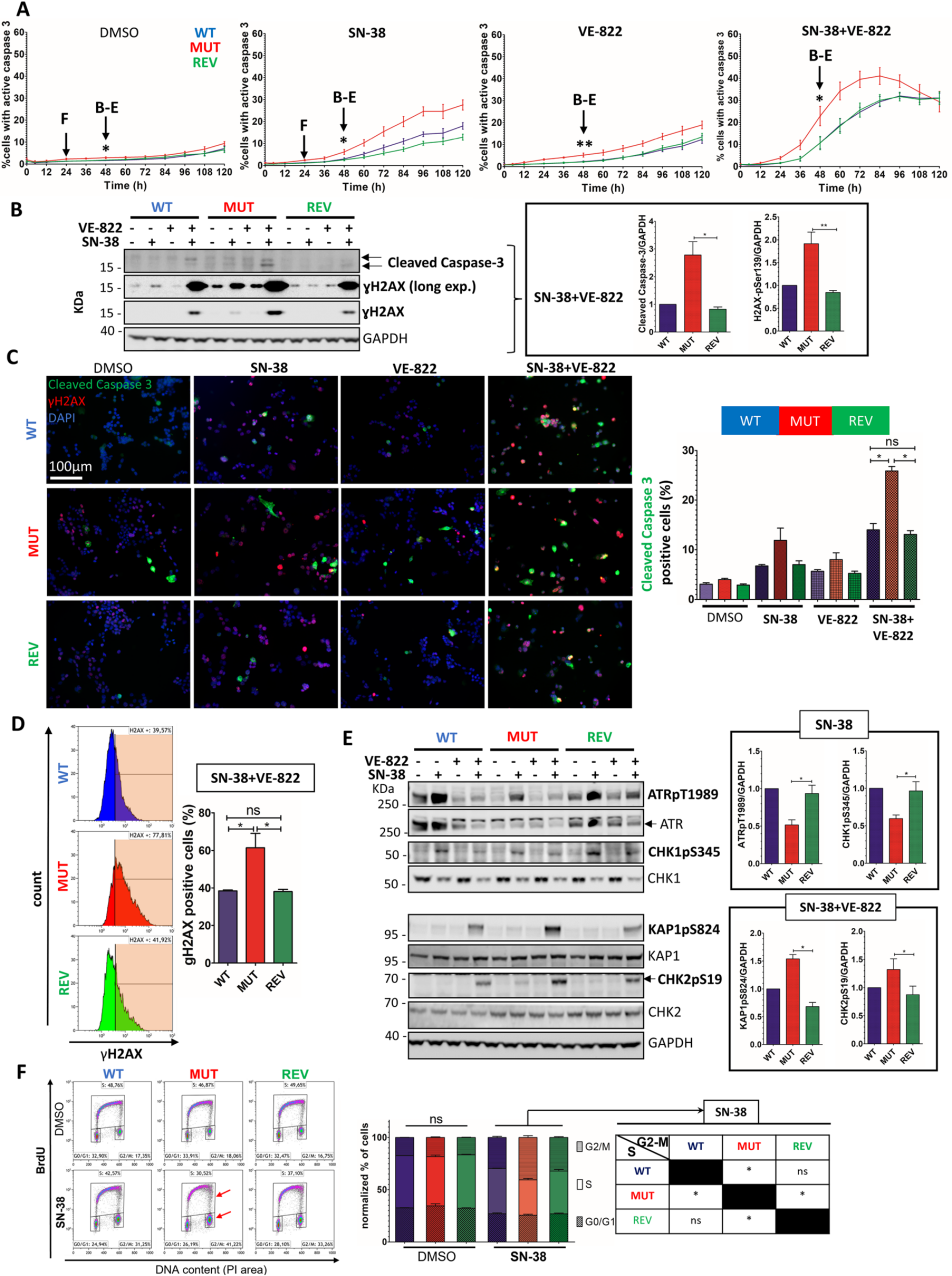
The ATR mutation sensitizes cells to SN-38 ± VE-822 through caspase-3 dependent apoptosis linked with increased DNA damage. (**A**) Cells were challenged with 1.5 nM SN-38 ± 50 nM VE-822 and assessed for caspase activity (Nexcelom) on the Celigo imager. The percentages of apoptotic cells were calculated. Error bars represent s.e.m from 5 independent replicates. → F (24 h) and → B-E (48 h) refers to the time points used in the experiments presented in (**B**)–(**F**). Mann–Whitney tests between MUT and WT/REV were applied on the 48 h timepoint. (**B**) Cleaved caspase-3 and γH2AX assessed by western blotting (48 h). GAPDH served as a loading control for each individual blot (see [Fig. S5]). A representative GAPDH panel is shown*.* The GAPDH-normalized quantifications relative to WT (SN-38 + VE-822) are calculated from 3 independent replicates. Mann–Whitney tests were applied. Full blots: [Fig. S5] (**C**) Immunofluorescence analysis of cleaved caspase-3 (green) and γH2AX (red) in cells treated with 1.5 nM SN-38 ± 50 nM VE-822 for 48 h. Cells were observed with a Zeiss Axio Imager (× 20), scale bar: 100 µm. An arbitrary threshold was set and percentage of cleaved caspase-3 positive cells was scored (n > 300 cells/sample). Error bars represent s.e.m from 3 experiments and Mann–Whitney tests were applied. (**D**) Flow cytometry assessment of γH2AX in cells challenged with SN-38 + VE-822. An arbitrary threshold was set (orange gates) and the percentage of positive cells was plotted. Error bars represent s.e.m from 3 independent experiments and Mann–Whitney tests were applied. (**E**) Western Blot analysis of total and phosphorylated ATR and CHK1 (ATR pathway) and KAP1 and CHK2 (ATM pathway) in cells challenged with 1.5 nM SN-38 ± 50 nM VE-822 (48 h). GAPDH served as a loading control for each individual blot (see [Fig. S5]). A representative GAPDH panel is shown. The GAPDH-normalized quantifications relative to WT (SN-38 or SN-38 + VE-822) are calculated from at least 3 independent replicates. Mann–Whitney tests were applied. ATR was re-stripped on the ATPpT1989 membrane. Full blots: [Fig. S5] (**F**) Cells were treated with 1.5 nM SN-38 for 24 h and pulsed with BrdU. Cells were processed on a Gallios (Beckman coulter). Debris and doublets were excluded, and 20,000 cells were analyzed per sample. Error bars are s.e.m from 3 independent experiments. Mann–Whitney tests were applied.

To better characterize the cytotoxic effects of the drugs, we assessed for caspase 3 activation using the Caspase 3–7 live kit (Nexcelom) on the Celigo, as a measure of apoptotic induction [Fig. 3A]. The basal levels of caspase activity in untreated cells were enhanced by both SN-38 and VE-822 treatments, in an ATR mutation-dependent manner. The combined (SN-38 + VE-822) treatment greatly enhanced apoptosis induction, especially in ATR mutant cells. Furthermore, assessing for the DNA content of the cells showed a dramatic increase in the sub-G1 population in response to SN-38 ± VE-822, especially in ATR mutant cells [Fig. S2B]. Moreover, the levels of cleaved-PARP—a product of caspases activation—followed the same pattern, thereby reinforcing the previous results [Fig. S2C]. Together, these data demonstrate that the ATR mutation is linked to an increased apoptosis induction by SN-38 ± VE-822. Because SN-38 induces RS, we suspected this apoptosis induction to be linked to DNA damage. To answer this question, we performed further analyses at the 48 h timepoint (↓B-E). Upon SN-38 + VE-822 treatment, increased levels of both cleaved caspase-3 and the DNA damage marker γH2AX were observed in the ATR mutant cells by WB [Fig. 3B], IF [Fig. 3C] and FACS [Fig. 3D], strongly correlating the ATR mutation with extensive apoptosis induction and DNA damage. We hypothesized that such phenotypes could be caused by a failure of the replication checkpoint in ATR mutant cells. We therefore quantified ATR-CHK1 and ATM-CHK2 activities in cells challenged by the drugs [Fig. 3E]. SN-38 alone induces a massive ATR-CHK1 pathway activation, which is significantly downregulated in ATR mutant cells (upper panel). Therefore, in presence of SN-38, ATR mutant cells fail to achieve a full activation of the ATR-CHK1 pathway. Furthermore, we demonstrated that this checkpoint failure was linked to a permissive replication progression despite the presence of SN-38 in ATR mutant cells challenged with SN-38 for 24 h [Fig. 3F]. Indeed, SN-38 tends to reduce the percentage of replicating cells while increasing in the G2/M, this being significantly exacerbated in the ATR mutant (red arrows). Moreover, while VE-822 totally abrogated the SN-38-induced ATR-CHK1 activation, ATM targets CHK2 and KAP1 were found hyper phosphorylated in response to SN-38 + VE-822, especially in ATR mutant cells [Fig. 3E] (lower panel). Altogether, these data suggest that the ATR mutation weakens the replication checkpoint of cells, leading to the accumulation of DNA damage and ultimately, to an enhanced apoptosis induction.

### The ATR signaling is the critical barrier protecting cells from ssDNA accumulation, RPA exhaustion and replication catastrophe during SN-38 treatment

**Figure 4:**
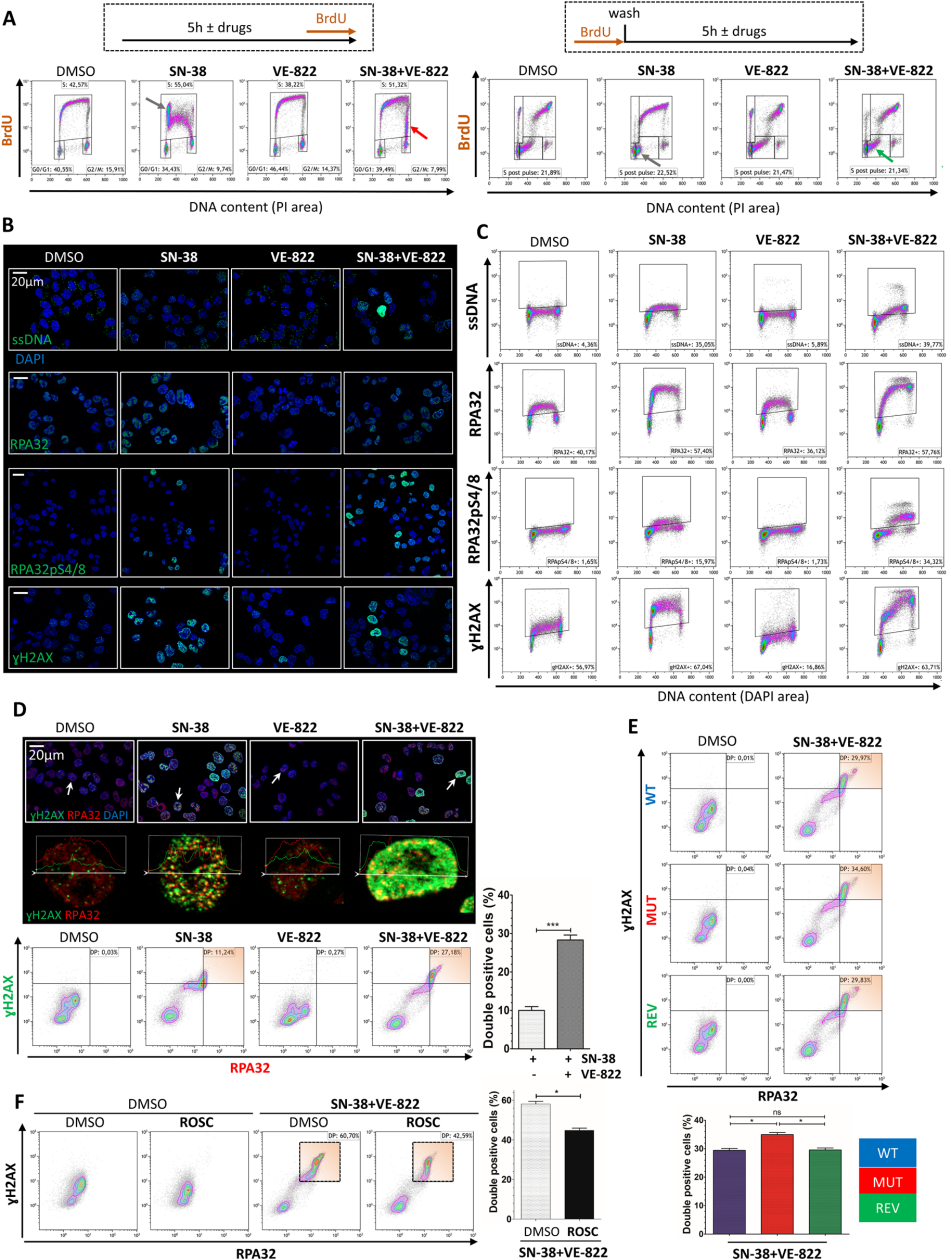
The ATR-CHK1 signaling is a critical barrier protecting cells from RPA exhaustion and replication catastrophe in cells challenged with SN-38. (**A**) WT cells were pulsed with BrdU after (left) or before (right) drug inductions (5 h; 40 nM SN-38 ± 2 µM VE-822). Doublets and debris were excluded and 20,000 cells were analyzed per sample. (**B**) Immunofluorescence analysis of WT cells challenged with drugs (same parameters). Target proteins were immunodetected, DNA was counterstained with DAPI and cells were observed with a Zeiss Axio Imager (apotome, × 63 objective) scale bars: 20 µm. (**C**) Flow cytometry analysis of WT cells challenged with drugs (same parameters). DAPI was used to stain DNA. 20,000 cells were analyzed per sample. ssDNA, RPA32, RPA32pS4-8 and γH2AX positive cells were arbitrary gated on the SN-38 + VE-822 quadrants and gates were pasted all conditions. (**D**) γH2AX and RPA32 were detected in immunofluorescence (upper panel). DAPI was used to counterstain DNA. Cells were observed with a Zeiss Axio Imager with apotome (× 63), scale bar: 20 µm or analyzed in FACS. Representative images from 3 independents experiments are shown. A representative cell of each sample is magnified (white arrow), without DAPI. Cross sections show the relative levels of RPA32 and γH2AX (“Profile” Zen Tool). For flow cytometry, RPA32 and γH2AX were detected, gates were set on the SN-38 quadrant and pasted on the other conditions. The increase of γH2AX intensity between SN-38 and SN-38 + VE-822 is highlighted in the orange gates. Percentages of double positive (orange gates) were plotted. The experiment was repeated 3 times. Mann–Whitney test was applied. (**E**) WT, MUT and REV cells were analyzed by flow cytometry. γH2AX and RPA32 double positive cells were gated (orange gates). The experiment was repeated 3 times. Mann–Whitney test was applied. (**F**) Flow cytometry analysis of WT cells challenged with SN-38 + VE-822 with or without roscovitine (ROSC, 20 µM). γH2AX and RPA32 were detected. Double-positive cells were gated (Orange) and plotted. Mann–Whitney test was applied.

As mentioned in the introduction, whether SN-38 could induce exhaustion of RPA and replication catastrophe (RC) is not known yet. Hence, we postulated that in response to SN-38, the ATR signaling serves as a critical barrier to replication progression and, potentially, RPA exhaustion and RC. In an attempt to answer these questions, we used higher and isotoxic (~ 20 IC_50_) concentrations of SN-38 (40 nM) and VE-822 (2 µM) to achieve optimal replication checkpoint activation and repression,as assessed by western blotting [Fig. S3A]. At these doses, a strong SN-38 (40 nM)-induced ATR-CHK1 activation is significantly impaired in ATR mutant cells, harboring more DNA damage. VE-822 (2 µM) completely suppressed the SN-38 -induced CHK1 phosphorylation. We then monitored cell cycle using complementary BrdU/PI labelling strategies during the acute response (5 h) to SN-38 and VE-822 [Fig. 4A]. We first studied the cell cycle distribution upon treatments (left panel). SN-38 induced an early-S cell cycle arrest (grey arrow) and reduces the BrdU incorporation of replicating cells. VE-822 by itself had no impact on cell cycle distribution. However, in combination with SN-38, VE-822 leads to a complete bypass of the SN-38-induced early-S arrest. Instead, we observed an accumulation of 4 N DNA BrdU positive cells (red arrow). We then studied the dynamics of replication progression by pulsing the cells with BrdU before drug inductions (“pre-drug pulse”, right panel), showing that VE822 completely restores the replication progression despite the presence of SN38 (grey and green arrows).

To answer if SN-38 and VE-822 induce an exhaustion of RPA, we assessed for ssDNA, RPA (total and phosphorylated at serines 4/8, as a DNA damage and checkpoint marker^32^) and the DNA damage marker γH2AX by immunofluorescence [Fig. 4B] and flow cytometry [Fig. 4C]. Globally, we showed that in replicating cells, SN-38 induces ssDNA linked with a massive RPA32 chromatin recruitment and phosphorylation at serines 4/8. γH2AX was also increased in replicating cells challenged with SN-38. VE-822 by itself did not induce any phenotype as compared to untreated controls, except a decrease of γH2AX levels in replicating cells. However, when coupled to SN-38, VE-822 greatly enhanced the generation of ssDNA and the recruitment/phosphorylation of RPA, in a replication dependent manner. Replication-dependent RPA recruitment to chromatin correlates with a dramatic increase in the γH2AX labeling. Interestingly, all these phenotypes (except for the phosphorylation of RPA at serines 4/8) were significantly enhanced in ATR mutant cells [Fig. S3B]. Thus, impaired ATR responses (endogenously or pharmacologically) in replicating cells challenged with SN-38 seem to trigger an exhaustion of RPA (massive chromatin recruitment), potentially driving cells into replication catastrophe (accumulation of DNA damage). To complete this analysis, we designed experiments to study the co-labellings of γH2AX and RPA32 coupling immunofluorescent and flow cytometry approaches in order to cover both sub-nuclear and pan-nuclear levels, respectively [Fig. 4D]. Representative cells pointed by white arrows are magnified below, with labelling-intensity cross sections. In normally growing cells, immunofluorescence highlighted few γH2AX foci spread throughout the nuclei. VE-822 did not affect these parameters. SN-38 led to an intense and co-occurring foci formation of γH2AX and RPA32. However, in a fraction of cells, SN-38 + VE-822 induced a strong, homogenous and pan-nuclear H2AX phosphorylation covering the RPA32 foci distribution, a classical phenotype of replication catastrophe (RC)^24,33^. Flow cytometry showed that SN-38 leads to the induction of γH2AX/RPA32 double positive cells. VE-822 by itself induced no phenotype as compared to the untreated control. However, when coupled to SN-38, VE-822 increased the percentage of double positive cells by enhancing the γH2AX intensity of RPA32 positive cells. This suggests that the RPA “protection threshold” was reached, inducing the accumulation of extensive DNA damage. Furthermore, IF and FACS experiments highlighted similar 53BP1pS1778 patterns, another DSB marker^34^ [Fig. S3C]. Together, these results demonstrate that SN-38 induces DNA damage (depleting RPA pools) which in turn lead to an ATR-dependent replication arrest. By abrogating this replication checkpoint, VE-822 contributes to the exhaustion of RPA as cells are forced to progress through replication, eventually leading to RC, characterized by extensive DNA damage. As we anticipated, these phenotypes were significantly enhanced in ATR mutant cells [Fig. 4E] hence giving a molecular explanation of their hypersensitivity to these drugs. A higher proportion of unchallenged ATR mutant cells showed increased levels of chromatin recruitment of RPA and H2AX phosphorylation [Fig. S3B], it is therefore likely that MUT cells are predisposed to the RPA exhaustion and RC induced by the SN-38 + VE-822 combination. Interestingly, roscovitine, a CDK inhibitor that prevents origin firing, partially protected cells from RPA exhaustion and DNA damage accumulation induced by the SN-38 + VE-822 combination [Fig. 4F]. This observation suggests that the negative regulation of origin firing by the ATR signaling is a critical mechanism targeted by VE-822, leading to the protection of cells from RPA exhaustion and RC in presence of SN-38.

As SN-38 + VE-822 leads to a 4 N-post replicative arrest of the cell cycle, we then wanted to identify the protein(s) involved in this post replication checkpoint. Indeed, having a complete understanding of compensatory checkpoint pathways that protect cells in the absence of ATR kinase activity is of high interest for future drug association in order to optimize SN-38/VE-822 efficiency, especially in ATR mutant cells.

### DNA-PK—but not the ATM-CHK2 axis—is primarily involved in the post replicative cell cycle arrest in response to SN-38 + VE-822

Amongst the DNA Damage response (DDR) major pathways, the three PI3K (ATR, ATM and DNA-PK) are known to hold concerted and non-redundant function in response to DNA lesions occurring during replication. Previous studies highlighted the relative roles played by these 3 branches of the DDR network in checkpoint responses to several DNA damaging agents, using combined siRNA and inhibitors of ATM, ATR (VE-821) and DNA-PK^32^. Here, we studied these processes in the context of the clinically relevant SN-38, using the most recent and specific ATR inhibitor VE-822. Therefore, in a replication stress context (SN-38) and with ATR being inhibited by VE-822, the ATM/CHK2 pathway and DNA-PK represent the two major checkpoint inducers that could account for the post replicative cell cycle arrest we observed. We used HCT116 cells knocked out for DNA-PKcs (DNA-PK^-/-^) and CHK2 (CHK2^-/-^) to study their putative involvement in the cell cycle arrest induced by SN-38 + VE-822.

**Figure 5:**
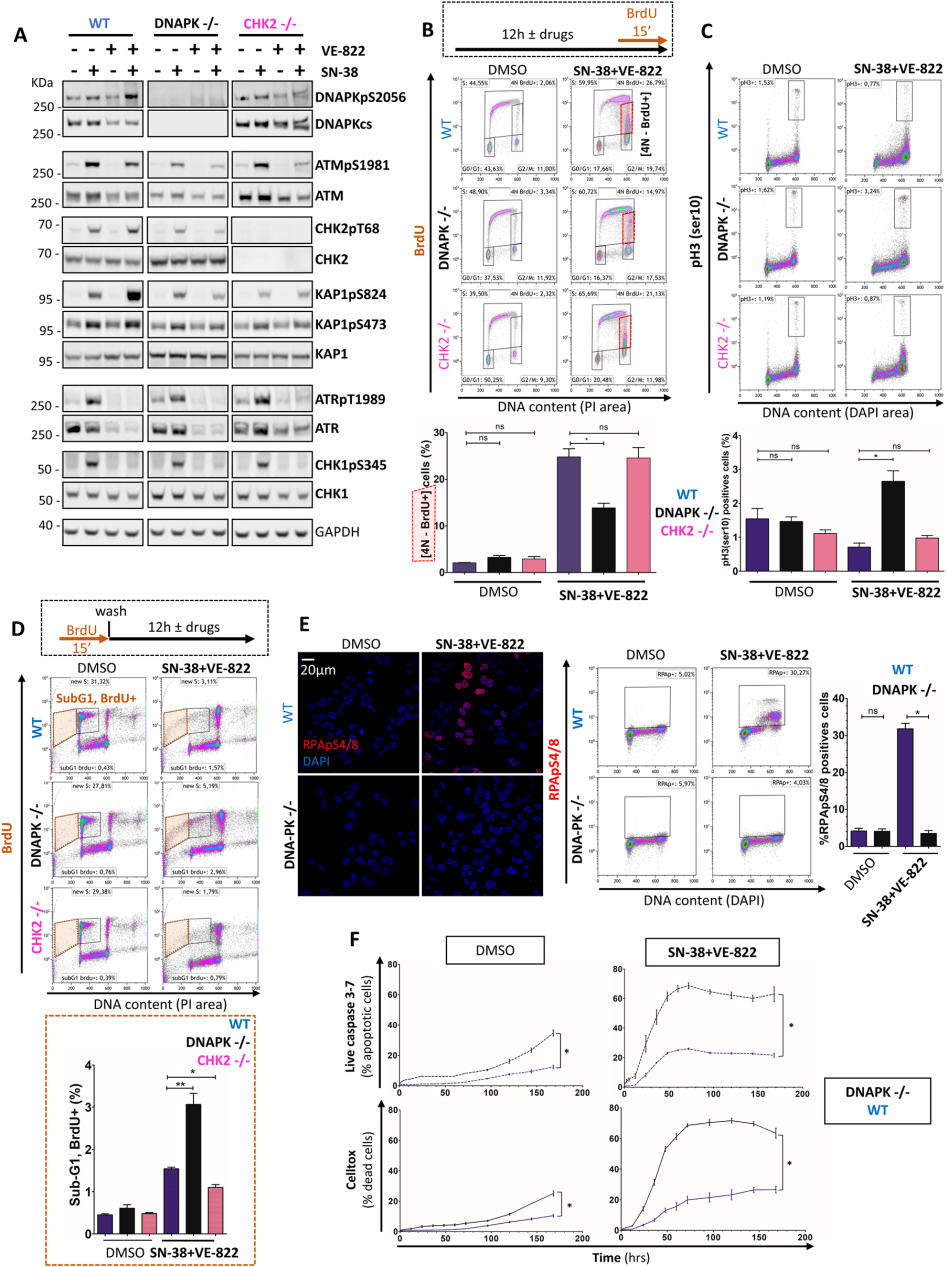
DNA-PK – but not the ATM-CHK2 pathway – activates a compensatory G2/M checkpoint in response to SN-38 when the ATR-CHK1 pathway is impaired. (**A**) Western Blots assessment of DNA-PK, ATM-CHK2 and ATR-CHK1 pathways in WT, DNAPK ^-/-^ and CHK2^-/-^ cells challenged with drugs (5 h; 40 nM SN-38 ± 2 µM VE-822). The experiments have been repeated at least 3 times and a representative blot of each protein is displayed.GAPDH served as a loading control for each individual blot (see [Fig. S5]). A representative GAPDH panel is shown. Displayed images are cropped from the same blots. Full blots: [Fig. S5] (**B**) Cell cycle analysis following drug induction (12 h). Cells were processed as described in [Fig. 4A]. [4 N DNA content – BrdU +] cells (red gates) were plotted. Error bars represent s.e.m from 3 independent experiments. Mann–Whitney tests were applied. (**C**) Flow cytometry assessment of phosphorylated H3 (ser10) as a marker of mitotic chromatin condensation. After drug induction (12 h), cells were harvested and prepared for flow cytometry. pH3 (ser10) positive cells were gated (debris and doublets excluded), and percentages were plotted on the graph. 20,000 cells were analyzed per sample. Error bars represent s.e.m from 3 independent experiments. Mann–Whitney tests were applied. (**D**) Flow cytometry analysis of cells pulsed with BrdU before the 12 h drug induction. BrdU + cells in Sub-G1 (< 2 N, orange gate) were gated and plotted; the experiment was repeated 3 times. Mann–Whitney tests were applied. (**E**) Flow cytometry and immunofluorescence analyses of RPA32 phosphorylation at serines 4/8 in WT or DNA-PK  ^−/−^ —cells. Cells were processed as described [Fig. 4B and C], respectively. For immunofluorescence, DNA was counterstained with DAPI and cells were observed at × 63, scale bar: 20 µm. For flow cytometry, DNA was counterstained with DAPI and 20,000 cells were analyzed per sample, debris and doublets excluded. RPA32pS4-8 positive cells were gated in WT and gates were pasted to DNAPK ^−/−^ . The percentages of positive cells were plotted. Errors bars represent the s.e.m from 3–10 independent replicates. Mann–Whitney tests were applied. (**F**) Cells were treated (40 nM SN-38 + 2 µM VE-822) and analyzed on the Celigo imager. The percentages of dead or apoptotic cells were assessed as described in [Figs. 2B, 3A]. Error bars represent s.e.m from 3 independent experiments. Mann–Whitney tests were applied.

While DNA-PK^-/-^ and CHK2^-/-^ cells completely lacked endogenous DNA-PK and CHK2 proteins, DNA-PKcs phosphorylation at serine 2056 as well as downstream targets of the ATM pathway (CHK2 Thr68, KAP1 Ser824, KAP1 Ser473) were as expected, found hyperphosphorylated by SN-38 + VE-822 in WT cells [Fig. 5A]. Besides, we also verified that DNA-PK^-/-^ and CHK2^-/-^ cells displayed a normal ATR-CHK1 pathway induction in response to SN-38, as compared to WT cells. In order to investigate if one of the two pathways is preferentially involved in the post-replicative cell cycle arrest induced by SN-38 + VE-822, we assessed cell cycle distribution. We observed that the [4 N, BrdU +] population was strongly depleted in DNA-PK^-/-^ cells treated with SN-38 + VE-822, but not in CHK2^-/-^ cells, suggesting that the post replicative cell cycle arrest is specifically abolished in the absence of DNA-PK [Fig. 5B]. Moreover, co-treating WT cells with the ATM inhibitor KU-55933 also failed to induce any noticeable difference in the post-replicative accumulation, reinforcing the hypothesis that this phenotype is independent of the ATM-CHK2 signaling [Fig. S4A]. In parallel, we observed a substantial increase in pH3-Ser10 phosphorylation (chromatin condensation marker) by flow cytometry [Fig. 5C] and western blotting [Fig. S4B] in DNA-PK^-/-^ cells treated by SN-38 + VE-822. Moreover, we set up an alternative BrdU pulse strategy to study the fate of DNA-PK^-/-^ cells going through mitosis in response to SN-38 + VE-822 [Fig. 5D]. [BrdU +  < 2 N] cells (cells that were in S phase during the BrdU pulse and entered Sub-G1/apoptosis during the SN-38 + VE-822 treatment) are twice more frequent in the absence of DNA-PK, but not CHK2. Furthermore, we observed by IF, flow cytometry and western blot [Figs. 5E, S4C] that DNA-PK^-/-^ cells fail to induce the phosphorylation of RPA (serines 4/8)that normally occurs when RPA chromatin recruitment reaches its peak in late-S/G2. We completed our study with a time course of cell death and apoptosis using the Celigo imager, assuming that DNAPK^-/-^ cells should be hypersensitive to SN-38 + VE-822, as they fail to induce their post replication checkpoint [Fig. 5F]. Following our predictions, cells lacking DNA-PK were greatly sensitized to the SN-38 + VE-822 combination. Besides, almost no lethality/apoptosis was observed at the 12 h timepoint, ruling out any potential bias in the cell cycle analyses that could be induced by cell death [Fig. 5 B, C]. Altogether, these data demonstrate that DNA-PK is a critical regulator of the post-replicative cell cycle arrest set up in G2/M in response to the SN-38 + VE-822 combination.

## Discussion

### The ATR mutation found in MSI + CRCs is critical during the cellular response to SN-38 and VE-822

RS is a hallmark of most cancers^4,35^By triggering the replication checkpoint, the ATR-CHK1 axis holds a pivotal role in the management of RS. Our results show that cells harboring the clinically relevant heterozygous mutation in ATR display a dysregulation of this checkpoint response, associated with higher endogenous replication stress and DNA damage. However, ATR mutant cells seem to somehow compensate for these phenotypes, as cell cycle and S-phase progression remain unaffected. Notably, the slowdown of replication forks observed in ATR mutant cells could likely be compensated by dormant origin firing, a phenotype observed during ATR inhibition^36^. Nevertheless, we rationally hypothesized that this ATR mutation, found in MSI + cancers, could be critical during drug response, especially for molecules known to be ATR-CHK1 inducers such as SN-38^37^. Indeed, our results highlighted a twofold-increased sensitivity of ATR mutant cells to SN-38. Moreover, a similar phenotype is observed when treating the cells with VE-822, a highly specific ATR inhibitor^38^. Furthermore, in agreement with recent publications, strong synergistic effects were highlighted between the two molecules^39^. In addition, we showed that the inhibition of the ATR pathway by VE-822 greatly potentiates the Caspase-3 dependent apoptosis induced by SN-38. Here again, the ATR mutation was linked to a twofold increased apoptotic induction in response to the drug combination SN-38 + VE-822. As VE-822 is currently in clinical trial (notably in combination with DNA damaging agents), we could expect significant benefits of these drug combinations especially in ATR mutated tumors. Interestingly, mutations in CHK1 exon7 have been reported as well and may induce similar phenotypes^40^. This could give the possibility to adapt posology depending on the tumor genotype, to reduce side effects.

### The ATR mutation predisposes cells to the exhaustion of RPA and replication catastrophe induced by the SN-38 + VE-822 combination

The RPA protein complex plays various critical roles to preserve genome integrity^41,42^. It has essential functions for the physical protection of single-stranded DNA and checkpoint activation^43^. It is notably involved in Nucleotide Excision Repair (NER) DNA repair pathway and various homologous recombination processes, including the Break-Induced Replication (BIR), the main DNA repair pathway managing the single ended DSBs induced by SN-38. It has been estimated that free RPA is in 6 to tenfold excess in normally growing cancer cells^25^. However, under replication stress conditions induced by antimetabolites and/or inhibition of the replication checkpoint, the free RPA pool can be exhausted as a result of a massive ssDNA induction during the uncoupling of helicases and polymerases^24^. This so called RPA exhaustion can drive the cells in a replication catastrophe (RC), characterized by irreversible DNA damage occurring at the ssDNA regions that can no longer be protected by RPA. Regulation of replication origin firing directly contributes to the management of soluble factors necessary for DNA replication. Thus, by inhibiting the firing of replication origins on a global scale, the activation of the ATR-CHK1 pathway helps cells to preserve their pools of soluble RPA^24^.

Even though Top1CCs induced by topoisomerase I inhibition do not cause helicase-polymerase uncoupling^26^, we show that it creates a propitious background to the exhaustion of RPA. Indeed, the generation of DSBs (as marked by γH2AX and 53BP1pS1778) likely promotes the generation of ssDNA via DNA resection by various exonucleases such as MRE11, EXO1, DNA2 or CtIP^44–46^. ATR-CHK1 activation is crucial to set-up the early-S replication arrest. As VE-822 allows cells to bypass this checkpoint and resume replication, most probably via unscheduled origin firing^36^, more DSBs are generated during the collisions between replication forks and Top1CCs, inducing higher levels of ssDNA as cells progress into replication. This exhausts the soluble pool of RPA and cells eventually reach a RC state, most likely in late-S/G2. This is consistent with our finding that the CDK inhibitor roscovitine protects cells from RPA exhaustion and with the known protective role of the ATR-CHK1 pathways in other replication stress contexts^24,25^. Moreover, we show that ATR mutant cells, which show higher basal levels of ssDNA, RPA chromatin recruitment and DNA damage, are predisposed to these detrimental outcomes, which likely explains their hypersensitivity to SN-38 and VE-822. RPA is abundantly expressed in the nucleus of eukaryotic cells and is overexpressed in numerous tumor types including lung, ovarian, and colorectal cancers^47^. Many efforts have been made to find RPA inhibitors in order to potentiate the anti-tumor activity effects of several molecules^48^. Indeed, the use of RPA inhibitors such as HAMNO^49^ or TDRL-505/551^50^, could lower the "RPA protection threshold", potentially increasing the efficiency of the SN-38 + VE-822 combination.

We observed that cells surviving the SN-38 + VE-822 combination complete replication and accumulate in a post-replicative state characterized by RPA hyperphosphorylation at serines 4/8 and high levels of DNA damage, notably DSBs marked by 53BP1pS1778^34^.

### Master regulator of the post-replication cell cycle arrest, DNA-PK is a potential co-target to achieve synthetic lethality with ATR inhibitors

Double-strand breaks (DSBs) are among the most toxic DNA lesions. At the cellular level, DSBs are mainly managed by the mutually exclusive HR (Homologous Recombination) and NHEJ (Non-Homologous End-Joining) pathways^51^. The accurate HR repair notably depends on ATM, the MRN complex and BRCA1/2 factors, while the repair by NHEJ (a far less reliable repair process) is mediated by the DNA-PK complex. During the canonical NHEJ, the catalytic subunit DNA-PKcs is recruited to the DSBs and auto-phosphorylates (Ser2056)^52^. We showed that the SN-38 + VE-822 combination triggers DNA-PK phosphorylation at Ser2056, which is correlated with the high levels of DNA damage resulting from the exhaustion of RPA. We showed that cells eventually get stopped in a post replicative state by DNA-PK, keeping them from entering mitosis. *A contrario*, DNA-PK^-/-^ cells fail to arrest in G2, progress through mitosis and are strongly sensitized to SN-38 + VE-822. Also, these processes were independent of the ATM-CHK2 pathway, as CHK2 deficient cells (as well as cells treated with the ATMi KU-55933) display a functional post-replication arrest.

Therefore, in response to SN-38 and in the absence of ATR kinase activity, DNA-PK seems to be preferentially involved in the post-replication checkpoint, preventing cells from progressing into a mitotic catastrophe. Indeed, DNA-PK deficient cells fail to phosphorylate serines 4/8 of RPA, which have been shown to protect cells from mitotic catastrophe following DNA damage^32^. Because of their non-redundant and coupled functions, ATR and ATM kinases were for a long time seen as putative co-targets for drug combination in cancer therapy^53^. Even so, our results suggest that targeting DNA-PK rather than ATM-CHK2 could better potentiate the ATR inhibition strategies, especially when coupled to a DNA damaging agent such as SN-38.

## Conclusions

In summary, we showed that the heterozygous ATR mutation is associated with high levels of endogenous replication stress, DNA damage and ATM-CHK2-KAP1 activities. Furthermore, the ATR mutation sensitizes cells to SN-38 and VE-822, both alone and combined. The increased apoptosis induction observed in mutant cells correlated with an impaired ATR-CHK1 induction and cell cycle arrest following SN-38 treatment. The highly synergistic combination SN-38 + VE-822 induces a massive chromatin recruitment of RPA in replicating cells, especially these harboring the ATR mutation. This exhaustion of soluble RPA leads a significant fraction of cells to a replication catastrophe state, while DNA-PK is responsible for their post-replicative arrest before the onset of mitosis. Thus, RPA and DNA-PK represent promising targets to potentialize the effects of ATR inhibitors coupled with DNA damaging agents. Furthermore, the significant impact of the ATR mutation in the response to SN-38 and VE-822 could open new doors in the management of ATR-mutated MSI + cancers.

## Material and methods

### Cell culture

HCT116 cells (NCI-DTP Cat# HCT-116, RRID:CVCL_0291) were cultured in McCoy’s 5A medium supplemented with 10 mM HEPES, 10 mM Sodium Pyruvate, 1% antibiotics and 10% FBS at 37 °C, 5% CO_2_. Each batch of cells used throughout the study was cultured for max. 3 months and passaged 2–3 times a week. Cells were tested negative for mycoplasma at every thawing and once a month during their culture periods. Cells were maintained in exponential growth phase. Before any drug treatments, cells were seeded at an equivalent density and allowed to attach overnight.

### ATR ZFN engineering & ATR genotyping

Sequence of ZFN binding site (underlined), Fok1 nuclease cutting site (**bold**), in the context of ATR exon 10 poly-A_10_ (*italics*): *AAAAAAAAAA*TACCTAGTCCAGTA**AAACTT**GGTGAGTGATTATGAC. Plasmids encoding Fok1 and ZFN targeting forward and reverse strands were obtained from Sigma-Aldrich (prod. Number CSTZFN-1KT, lot: 03151110MN). Primers targeting flanking sequences of the poly-A/cutting site were ordered from Sigma-Aldrich. Primers sequences are: F: 5'-TTCCCCAGAGATAAAGTCAAAGA-3'; R: 5'-CCTGTAATTTTTCAAGGCTTCAG-3' (amplicon length: 368 bp). Briefly, ATR MUT cells were prepared for transfection, and co-transfected with ZFN plasmids, according to manufacturer’s instructions. Cloning dilution was performed in 96 well plates and revertant clones were identified by PCR (i.e. WT/WT ATR clones generated from MUT cells). To do so, DNA was extracted (NucleoSpin, Macherey Nagel, 740952.250). The PCR program was 98 °C, 30 s, followed by 35 cycles of the following sequence {98 °C, 10 s; 55 °C 15 s; 72 °C, 15 s}. Sequencing was performed by Eurofins Genomics. ATR genotypes of clones were monitored throughout the study to ensure that they remained stable.

### Statistical analysis

Statistical analysis was performed on GraphPad Prism 5. Error bars on graphs are s.e.m from at least 3 independent biological replicates, unless stated otherwise. One-tailed Mann–Whitney tests significances are: *: *p* < 0.05; **: *p* < 0.01; ***: *p* < 0.001.

### Sulforhodamine B survival assays (SRB assay)

1500 cells were seeded in 96-well plates and treated in triplicate. After 72 h, cells were fixed overnight in 10% Tri-Chloroacetic Acid. Cells were washed 3 times with Milli-Q water and 50µL of 1% acetic acid-0.4% SRB were added for 30 min. Cells were washed 3 times in 1% acetic acid and SRB was resuspended in 100µL of 10 mM Tris-Base. OD was measured at 560 nm. The survival fraction (*SF*) (%) at each point was calculated as follows:

$$SF \left( \% \right) = \frac{{average\,OD\,\left( {Sample\,triplicate} \right)}}{{average\,OD\, \left( {Untreated\,triplicate} \right)}} \times 100$$

Survival curves were plotted using GraphPad Prism*.* The experiments were replicated three times, standardized curves and IC_50_ were generated using non-linear regression in *prism 5*.

### Antibodies and resources

The primary antibodies used throughout the study were: Cleaved Caspase 3 (Cell Signaling Technology Cat# 9661, RRID:AB_2341188), γH2AX (Cell Signaling Technology Cat# 9718, RRID:AB_2118009), ATRpT1989 (GeneTex Cat# GTX128145, RRID:AB_2687562), ATR (Cell Signaling Technology Cat# 2790, RRID:AB_2227860), CHK1pS345 (Cell Signaling Technology Cat# 2348, RRID:AB_331212), CHK1 (Cell Signaling Technology Cat# 2360, RRID:AB_2080320), KAP1pS824 (Cell Signaling Technology Cat# 4127, RRID:AB_2209906), KAP1 (Abcam Cat# ab22553, RRID:AB_447151), CHK2pS19 (Cell Signaling Technology Cat# 2666, RRID:AB_330057), CHK2 (Cell Signaling Technology Cat# 2662, RRID:AB_2080793), GAPDH (Abcam Cat# ab8245, RRID:AB_2107448), BrdU (Bio-Rad Cat# OBT0030, RRID:AB_609568), ssDNA (Millipore Cat# MAB3034, RRID:AB_94645), RPA32 (Abcam Cat# ab2175, RRID:AB_302873), RPA32pS4/8 (Bethyl Cat# A300-245A, RRID:AB_210547), β-tubulin (Abcam Cat# ab21057, RRID:AB_727043), 53BP1pS1778 (Cell Signaling Technology Cat# 2675, RRID:AB_490917), DNA-PKpS2056 (Abcam Cat# ab18192, RRID:AB_869495), DNA-PKcs (Cell Signaling Technology Cat# 4602, RRID:AB_10692482), ATMpS1981 (Cell Signaling Technology Cat# 13050, RRID:AB_2798100), ATM (Cell Signaling Technology Cat# 2873, RRID:AB_2062659), CHK2pT68 (Cell Signaling Technology Cat# 2197, RRID:AB_2080501), KAP1pS473 (Abcam Cat# ab133225, RRID:AB_11157160).

The secondary antibodies were: goat anti-mouse IgG—HRP (SouthernBiotech Cat# 1030-05, RRID:AB_2619742), goat anti-rabbit IgG—HRP (SouthernBiotech Cat# 4055-05, RRID:AB_2795980), goat anti-rat IgG FITC (SouthernBiotech Cat# 3030-02, RRID:AB_2795818), goat anti-rabbit Alexa 488 (Thermo Fisher Scientific Cat# A-11008, RRID:AB_143165), goat anti-mouse Alexa 555 (Thermo Fisher Scientific Cat# A-21422, RRID:AB_2535844), goat anti-mouse IgG1 Pe-Cy7 (Thermo Fisher Scientific Cat# 25-4015-82, RRID:AB_11150243).

The commercial kits used were: CellTox Green Cytotoxicity assay (Promega, G8741), ViaStain Live caspase 3/7 detection for 2D/3D culture (Nexcelom, CSK-V0002-1), Apoptosis, DNA Damage and Cell Proliferation Kit (BD Biosciences, 562253).

### Western blotting

10^6^ cells were seeded in 6-well plates. After drug treatments, cells were trypsinized and washed with ice-cold PBS. Proteins were extracted in M-PER extraction buffer containing 1 mM EDTA and a proteases/phosphatases inhibitor cocktail (Thermo Fisher) (1000 rpm agitation, RT, 15 min). Debris were spun down (20 min, 13,000 rpm, 4 °C) and Protein extracts were dosed (Pierce BCA Kit, Thermo Fisher). Equivalent amounts of proteins were then diluted into loading buffer, sample reducing agent (Thermo Fisher) and Milli-Q water. Migration was performed using 3–8% (Tris–acetate buffer) and 4–12% (Bis–Tris buffer) acrylamide gradient pre-cast gels (Thermo Fisher), according to manufacturer’s instruction. Transfer was performed on nitrocellulose membranes (iBlot, Thermo Fisher). Membranes were saturated in TBS-Tween 0.1%-5% non-fat milk and incubated (overnight, 4 °C) with primary antibodies diluted in TBS-0.1%Tween-5%BSA (Sigma-Aldrich). Membranes were washed 3 × 10 min in TSB-Tween 0.1% and incubated (1 h, RT) with peroxidase-conjugated secondary antibodies (Southern-Biotech), diluted at 1:3000 in TBS-0.1%Tween-5% milk. Membranes were washed 3 × 10 min in TSB-0.1%-Tween. ECL RevelBlot Plus was used and membranes were revealed in a G:BOX (Ozyme) using the Syngene software. Non-saturating blots were displayed. Full length blots are available [Fig. S5].

### Immunofluorescence

Cells were grown on Poly-D-Lysine-coated 14 mm glass coverslips in 6-well plates. After drug treatments, cells were washed in PBS and fixed in 4% formaldehyde (Thermo Fisher) (15 min, RT). Cells were permeabilized in PBS-0.2% Triton -X-100 (10 min, RT), before being saturated in a PBS-0.1% Tween-2% BSA blocking buffer (1 h, RT). The coverslips were incubated with primary antibodies (overnight, 4 °C). Coverslips were washed 3 × 10 min in PBS-0.1% Tween and incubated with the secondary antibodies (Thermo Fisher) diluted at 1:200 in blocking buffer (1 h, RT). Coverslips were washed 3 × 10 min. Nuclei were counterstained with 5 µg/mL DAPI (2 min, RT). Coverslips were rinsed with PBS and mounted on Superfrost slides in ProLong Gold Antifade mountant (Thermo Fisher). Cells were observed on a Zeiss Axio Imager M2 microscope with Apotome. Pictures were processed and analyzed and scored with *Zen Blue* dedicated software.

### DNA fiber assay

0.25 × 10^6^ cells were seeded in 12-well plates. Cells were sequentially pulsed with 15 µM 5-Iodo-2’-deoxyuridine (IdU) (15 min) and 200 µM 5-Chloro-2’-deoxyuridine (CldU) (15 min). Cells were then trypsinized, washed and diluted in PBS (0.5–1 × 10^6^ cells/mL). 2 µL of cell suspension were dropped on the edge of Superfrost slides. After 2–5 min drying at RT, 7 µL of DNA Spreading buffer (200 mM Tris–HCl pH 7.5, 50 mM EDTA, 0.5% SDS) were mixed with the drying cell suspension drops. After 2–5 min drying at RT, slides were inclined (15–30°) in order to spread the DNA fibers, which were then fixed for 10 min in a 3:1 methanol/acetic acid solution. Slides were washed in distillated water and DNA was denatured for 1 h in 2.5 M HCl. Slides were washed and saturated in a blocking buffer (PBS-1% BSA 0.1% Tween-20) (1 h, RT). Slides were incubated 1 h with a mix of Anti-IdU (mouse anti-BrdU clone B44, Becton Dickinson, 347580) and Anti-CldU (rat anti-BrdU clone BU1/75, AbCys SA, ABC117 7513) both diluted at 1/20 in blocking buffer at 37 °C. Slides were washed 5 × 2 min in a washing buffer (PBS-0.1% Tween-20) and incubated with a mix of the following secondary antibodies: chicken-anti rat Alexa 488 (Molecular probes, A2147) and chicken anti-mouse IgG1 Alexa 546 (Molecular Probes, A21123) both diluted at 1/50 in blocking buffer (30 min, 37 °C). Slides were washed 5 × 2 min in PBS-0.1% Tween-20 and incubated with a mouse IgG2a anti-ssDNA (Chemicon, MAB3034) antibody diluted at 1/500 in blocking buffer (30 min, 37 °C). Slides were washed 5 × 2 min in washing buffer and incubated with a goat anti-mouse IgG2a Alexa 647 secondary antibody (Molecular Probes, A21241), diluted at 1/50 in blocking buffer (30 min, 37 °C). Slides were washed 5 × 2 min in washing buffer. Slides were then mounted under 24 × 50 mm coverslips with 30 µL of ProLong Gold Antifade mountant (Thermo Fisher). 20 representative pictures of each condition were taken on a Leica DM6000 microscope (× 40 objective). The experiment was performed three times independently, and a total of 600 (3 × 200) signals (CldU tracks following IdU track) were randomly measured using ImageJ. Mann–Whitney tests were used to determine significance.

### Flow cytometry

#### Chromatin bound proteins (RPA32, RPApS4-8, γ-H2AX, 53BP1pS1778)

1 × 10^6^ cells were seeded in 6-well plates. After drug treatments, cells were trypsinized, pooled with their supernatants and washed with cold PBS. Fixation and immunodetection were performed according to Forment & Jackson^54^. Cells were incubated overnight in PBS-1 µg/mL DAPI, 100 µg/mL RNase A and analyzed on a Gallios Flow Cytometer (Beckman Coulter). Forward Scatter/Side Scatter based debris exclusion was set up, and doublets were excluded using the DAPI Height/DAPI Area graphs. 20,000 cells were analyzed per sample on Kaluza dedicated software.

#### Cell cycle analysis: BrdU/PI

Cells were seeded in 6-well plates. After drug treatments, cells were trypsinized before being pooled with their supernatants and rinsed with cold PBS. Cells were fixed by adding ethanol to a final concentration of 70% (overnight, 4 °C). Cells were digested in 30 mM HCl, 0.5 mg/mL pepsin (20 min at 37 °C), DNA was denatured in 2 N HCl (20 min at RT). Cells were rinsed with PBS and resuspended in 250µL of BU buffer (PBS- 2% goat serum- 0.5% HEPES- 0.5% Tween-20) containing the anti-BrdU antibody (1:25, 1 h, RT). Nuclei were pelleted and resuspended into 200 µL of BU buffer containing the secondary antibody (1:50, 30 min, RT). Cells were rinsed and resuspended in staining buffer (PBS-25 µg/mL Propidium Iodide) and left overnight at 4 °C. Nuclei were analyzed on a Gallios Flow cytometer. A FSC/SSC graph was used to gate out debris. PI Height/PI Area graph was used to gate out doublets. 20,000 cells were analyzed for each sample on Kaluza.

### Apoptosis and cell death quantification on living cells (celigo imaging cytometer)

All experiments were performed in 96-well plates (Thermo, 165,305). 1250 cells (100 µL) were seeded per well and left overnight. Cells were treated with a mixture of drugs and live stains (Live Caspase-3/7 or CellTox green reagent, at recommended dilutions). The combined use of these two kits allowed us to study the early (Caspase-3/7 activation) and later (CellTox Green) cell death events. An untreated proliferation control with no live stain was assayed for each clone in every experiment. The green fluorescence was analyzed on the Celigo in living cells at the indicated timepoints. Each experiment was repeated 5 times, and curves were plotted using GraphPad prism (errors bars: s.e.m). Acquisition and analysis settings were the following. Acquisition and analysis modes of the Celigo were set to “dead/total”. Exposure time was arbitrarily set to 100,000. General settings used for the analysis were: Use well mask: 1; Well Mask: 95%; Well shape override:0. Dead frame settings: Algorithm: 1 (fluorescence); Intensity threshold: 3; Precision: 2; BFsharpen: 0; FilterSize: 10; Background correction: 0; Separate touching objects: 1; Cell area range: 20–10,000; Cell intensity range: 20–255; Cell Smoothness min: 0; Cell aspect ratio min: 0. Total cells frame settings: Algorithm: 0 (bright field); Intensity threshold: 4; Precision: 2; BFsharpen: 0; FilterSize: 10; Background correction: 1; Separate touching objects: 0; Cell area range: 50–10,000; Cell intensity range: 25–255; Cell Smoothness min: 0; Cell aspect ratio min: 0.

## Supplementary Information

Supplementary Information.

## Data Availability

Raw data were generated at the IRCM – INSERM U1194. Derived data supporting the findings of this study are available from the corresponding authors A.C and T.E. on request.

## References

[1] Aparicio J (2005). FOLFOX alternated with FOLFIRI as first-line chemotherapy for metastatic colorectal cancer. Clin. Colorectal Cancer.

[2] Mishima H, Ikenaga M, Yasui M (2011). Safety and efficacy of FOLFOX and FOLFIRI in elderly patients with colorectal cancer. Nihon Rinsho.

[3] Da-Re C, Halazonetis TD (2015). DNA replication stress as an Achilles' heel of cancer. Oncotarget.

[4] Macheret M, Halazonetis TD (2015). DNA replication stress as a hallmark of cancer. Annu. Rev. Pathol..

[5] Kitao H (2018). DNA replication stress and cancer chemotherapy. Cancer Sci..

[6] Cimprich KA, Cortez D (2008). ATR: an essential regulator of genome integrity. Nat. Rev. Mol. Cell Biol..

[7] Shiotani B, Zou L (2009). ATR signaling at a glance. J. Cell. Sci..

[8] Blackford AN, Jackson SP (2017). ATM, ATR, and DNA-PK: The Trinity at the Heart of the DNA Damage Response. Mol. Cell.

[9] Saldivar JC, Cortez D, Cimprich KA (2017). The essential kinase ATR: ensuring faithful duplication of a challenging genome. Nat. Rev. Mol. Cell Biol..

[10] Zou L, Elledge SJ (2003). Sensing DNA damage through ATRIP recognition of RPA-ssDNA complexes. Science.

[11] Kumagai A, Lee J, Yoo HY, Dunphy WG (2006). TopBP1 activates the ATR-ATRIP complex. Cell.

[12] Mordes DA, Glick GG, Zhao R, Cortez D (2008). TopBP1 activates ATR through ATRIP and a PIKK regulatory domain. Genes Dev.

[13] Lee YC, Zhou Q, Chen J, Yuan J (2016). RPA-binding protein ETAA1 Is an ATR activator involved in DNA replication stress response. Curr Biol.

[14] Goto H, Kasahara K, Inagaki M (2015). Novel insights into Chk1 regulation by phosphorylation. Cell Struct. Funct..

[15] Enders GH (2008). Expanded roles for Chk1 in genome maintenance. J. Biol. Chem..

[16] Zhang Y, Hunter T (2014). Roles of Chk1 in cell biology and cancer therapy. Int. J. Cancer.

[17] Toledo LI, Murga M, Fernandez-Capetillo O (2011). Targeting ATR and Chk1 kinases for cancer treatment: a new model for new (and old) drugs. Mol. Oncol..

[18] Karnitz LM, Zou L (2015). Molecular pathways: targeting ATR in cancer therapy. Clin. Cancer Res..

[19] Rundle S, Bradbury A, Drew Y, Curtin NJ (2017). Targeting the ATR-CHK1 axis in cancer therapy. Cancers (Basel).

[20] Pourquier P, Pommier Y (2001). Topoisomerase I-mediated DNA damage. Adv Cancer Res.

[21] Pommier Y (2006). Topoisomerase I inhibitors: camptothecins and beyond. Nat. Rev. Cancer.

[22] Xu Y, Her C (2015). Inhibition of topoisomerase (DNA) I (TOP1): DNA damage repair and anticancer therapy. Biomolecules.

[23] Sakasai R, Iwabuchi K (2016). The distinctive cellular responses to DNA strand breaks caused by a DNA topoisomerase I poison in conjunction with DNA replication and RNA transcription. Genes Genet Syst..

[24] Toledo LI (2013). ATR prohibits replication catastrophe by preventing global exhaustion of RPA. Cell.

[25] Toledo L, Neelsen KJ, Lukas J (2017). Replication catastrophe: when a checkpoint fails because of exhaustion. Mol. Cell.

[26] Patro BS, Frohlich R, Bohr VA, Stevnsner T (2011). WRN helicase regulates the ATR-CHK1-induced S-phase checkpoint pathway in response to topoisomerase-I-DNA covalent complexes. J. Cell Sci..

[27] Lewis KA (2005). Heterozygous ATR mutations in mismatch repair-deficient cancer cells have functional significance. Cancer Res..

[28] Lewis KA (2007). Mutations in the ataxia telangiectasia and rad3-related-checkpoint kinase 1 DNA damage response axis in colon cancers. Genes Chromosom. Cancer.

[29] Sanchez Y (1997). Conservation of the Chk1 checkpoint pathway in mammals: linkage of DNA damage to Cdk regulation through Cdc25. Science.

[30] Mailand N (2000). Rapid destruction of human Cdc25A in response to DNA damage. Science.

[31] Nilsson I, Hoffmann I (2000). Cell cycle regulation by the Cdc25 phosphatase family. Prog. Cell Cycle Res..

[32] Ashley AK (2014). DNA-PK phosphorylation of RPA32 Ser4/Ser8 regulates replication stress checkpoint activation, fork restart, homologous recombination and mitotic catastrophe. DNA Repair (Amst).

[33] Buisson R, Boisvert JL, Benes CH, Zou L (2015). Distinct but concerted roles of ATR, DNA-PK, and Chk1 in countering replication stress during S phase. Mol. Cell.

[34] Lee JH, Cheong HM, Kang MY, Kim SY, Kang Y (2009). Ser1778 of 53BP1 Plays a role in DNA double-strand break repairs. Korean J. Physiol. Pharmacol..

[35] Hanahan D, Weinberg RA (2011). Hallmarks of cancer: the next generation. Cell.

[36] Moiseeva T (2017). ATR kinase inhibition induces unscheduled origin firing through a Cdc7-dependent association between GINS and And-1. Nat. Commun..

[37] Flatten K (2005). The role of checkpoint kinase 1 in sensitivity to topoisomerase I poisons. J. Biol. Chem..

[38] Weber AM, Ryan AJ (2015). ATM and ATR as therapeutic targets in cancer. Pharmacol. Ther..

[39] Josse R (2014). ATR inhibitors VE-821 and VX-970 sensitize cancer cells to topoisomerase i inhibitors by disabling DNA replication initiation and fork elongation responses. Cancer Res..

[40] Bertoni F (1999). CHK1 frameshift mutations in genetically unstable colorectal and endometrial cancers. Genes Chromosom. Cancer.

[41] Bhat KP, Cortez D (2018). RPA and RAD51: fork reversal, fork protection, and genome stability. Nat. Struct. Mol. Biol..

[42] Zou Y, Liu Y, Wu X, Shell SM (2006). Functions of human replication protein A (RPA): from DNA replication to DNA damage and stress responses. J. Cell Physiol..

[43] Oakley GG, Patrick SM (2010). Replication protein A: directing traffic at the intersection of replication and repair. Front Biosci. (Landmark Ed).

[44] Shiotani B, Zou L (2009). Single-stranded DNA orchestrates an ATM-to-ATR switch at DNA breaks. Mol. Cell.

[45] Limbo O, Porter-Goff ME, Rhind N, Russell P (2011). Mre11 nuclease activity and Ctp1 regulate Chk1 activation by Rad3ATR and Tel1ATM checkpoint kinases at double-strand breaks. Mol. Cell Biol..

[46] Shibata A (2014). DNA double-strand break repair pathway choice is directed by distinct MRE11 nuclease activities. Mol. Cell.

[47] Jekimovs C (2014). Chemotherapeutic compounds targeting the DNA double-strand break repair pathways: the good, the bad, and the promising. Front Oncol..

[48] Gavande NS (2016). DNA repair targeted therapy: The past or future of cancer treatment?. Pharmacol. Ther..

[49] Glanzer JG (2014). RPA inhibition increases replication stress and suppresses tumor growth. Cancer Res..

[50] Mishra AK, Dormi SS, Turchi AM, Woods DS, Turchi JJ (2015). Chemical inhibitor targeting the replication protein A-DNA interaction increases the efficacy of Pt-based chemotherapy in lung and ovarian cancer. Biochem. Pharmacol..

[51] Sonoda E, Hochegger H, Saberi A, Taniguchi Y, Takeda S (2006). Differential usage of non-homologous end-joining and homologous recombination in double strand break repair. DNA Repair (Amst).

[52] Davidson D, Amrein L, Panasci L, Aloyz R (2013). Small Molecules, Inhibitors of DNA-PK, Targeting DNA Repair, and Beyond. Front Pharmacol..

[53] Kantidze OL, Velichko AK, Luzhin AV, Petrova NV, Razin SV (2018). Synthetically Lethal Interactions of ATM, ATR, and DNA-PKcs. Trends Cancer.

[54] Forment JV, Jackson SP (2015). A flow cytometry-based method to simplify the analysis and quantification of protein association to chromatin in mammalian cells. Nat. Protoc..

