# Supplementary material for: A clinically relevant heterozygous ATR mutation sensitizes colorectal cancer cells to replication stress
Source: Sci Rep. 2022 Mar 31;12:5422. doi: 10.1038/s41598-022-09308-4 (PMC8971416; doi:10.1038/s41598-022-09308-4)

Supplementary information to:

A clinically relevant heterozygous ATR mutation sensitizes colorectal cancer cells to replication stress

Tom Egger[1,2,*], Benoît Bordignon[1,3] and Arnaud Coquelle[1,*]

[1] IRCM, Institut de Recherche en Cancérologie de Montpellier, INSERM U1194, Université de Montpellier, Institut régional du Cancer de Montpellier, Montpellier, F-34298, France

[2] (current address) Institut de Génétique Humaine, UMR9002, CNRS-Université de Montpellier, 34396, Montpellier, France

[3](current address) Montpellier Ressources Imagerie, BioCampus, University of Montpellier, CNRS, INSERM, Montpellier, France

* corresponding authors:

Tom Egger: tom.egger@hotmail.fr

Arnaud Coquelle: arnaud.coquelle@inserm.fr

Figure S1: The ATR mutation induces endogenous DNA damages without impacting cell cycle

A) Western blot analysis of CDC25A. GAPDH was used as a loading control. Quantifications relative to WT were calculated from 3-5 independent replicates. Mann-Whitney tests were applied. Full blots: [Fig. S5]. **B)** Western blot analysis of ɣH2AX. Total H2AX and GAPDH were used as a loading controls. Quantifications relative to WT were calculated from 3-5 independent replicates. Mann-Whitney tests were applied. Full blots: [Fig. S5]. **C)** BrdU/PI Flow cytometry assessment of cell cycle **distribution**. Cells were pulsed with BrdU and immediately fixed in cold ethanol. BrdU immunodetection was performed and DNA was stained with PI. Flow cytometry was performed on a Gallios flow cytometer (Beckman Coulter). Debris and doublets were excluded, and 20,000 events were analyzed per condition. The percentage of cells in each phase of the cell cycle was calculated. The plot summarizes the data from 3 biological replicates. Mann-Whitney tests were applied between each clone for each cell cycle phase. **D)** Flow cytometry assessment of cell cycle and replication **progression** using a BrdU/PI pulse-chase strategy. Cells were pulsed with BrdU before a 5 h chase. Cells were then fixed in cold ethanol, BrdU was immunodetected and DNA was stained with propidium iodide (PI). Flow cytometry was performed on a Gallios flow cytometer (Beckman Coulter). Debris and doublets were excluded, and 20,000 events were detected per condition. The percentage of cells that entered S-phase during the 5 h post BrdU was quantified (red gates). The experiments were repeated three times and the results were plotted. Mann-Whitney tests were applied.

Figure S2: ATR mutation enhances apoptosis induction in response to SN-38+VE-822

A) Upper panel: SRB assays performed in WT HCT116 in presence of hydroxyurea (HU), aphidicolin (APH) and taxol. Survival curves were plotted using GraphPad Prism from two independent replicates for APH and HU, or a single experiment (mean of triplicates) for taxol. Error bars represent s.e.m. **Lower panel:** Synergy plots for 8-doses combinations of VE-822 with HU, APH or taxol in WT HCT116 cells. Plot was obtained using the Mac Synergy plugin in Microsoft Excel, doses of each drug are displayed in (X) and (Y). Synergy (%) is displayed in (Z). The experiment was repeated two times (HU, APH) and once (taxol). **B**) Sub-G1 analysis following 48 h induction with 1.5 nM SN-38 ± 50 nM VE-822. DNA content was assessed using DAPI and n=20,000 cells were analyzed per sample on a Gallios flow cytometer. DAPI is displayed on a log scale. **C**) Cleaved-PARP analysis using the DNA damage and proliferation kit (BD bioscience). n=20,000 cells were analyzed per sample, debris and doublets excluded. Quantifications for each protocol are presented below, errors bars show the s.e.m from three independent experiments, and Mann-Whitney tests were applied.

Figure S3: The ATR mutation potentiates the induction of ssDNA, RPA exhaustion and DNA damages in a cell cycle independent manner

A) Western blot analysis of ATR, CHK1 and H2AX phosphorylation on Thr1989, Ser345, and Ser139 in cells challenged with the same drug treatments as in [Fig. 4] (5 h; 40 nM SN-38 ± 2 µM VE-822). GAPDH served as a loading control for each individual blot (see [Fig. S5]). A representative GAPDH panel is shown. Mann-Whitney tests were applied. The GAPDH-normalized quantifications relative to WT (SN-38) are calculated from at least 3 independent replicates. Full blots: [Fig. S5]. **B**) Extended data from [Fig. 4C] including WT, MUT and REV cells. Percentages of cells positive for either ssDNA, RPA32/2, RPApS4-8 or γH2AX were plotted in adjacent graphs. Error bars represent variabilities from 3-10 independent experiments and Mann-Whitney tests were applied. **C**) Immunofluorescence and FACS analysis of 53BP1pS1778 (double strand break marker) and RPA32 in WT cells (scale bar: 20µm). Same protocols and analysis settings as [Fig. 4D]. A Mann-Whitney test was applied.

Figure S4: DNA-PK – but not the ATM-CHK2 pathway – activates a compensatory G2/M checkpoint in response to SN-38 when the ATR-CHK1 checkpoint is impaired

A) Cell cycle analysis following 12h induction with 40 nM SN-38 + 2 µM VE-822 in presence or absence of 10 µM KU-55933 (ATM inhibitor). After drug treatments, cells were pulsed with BrdU (30 µM, 15 min), harvested, prepared and analyzed on a Gallios flow cytometer. Adjacent western blots show the decreased phosphorylation of ATM-CHK2 targets, including H2AX, in the same experimental settings. Displayed images are cropped from the same blot. GAPDH was used as a loading control. Full blots: [Fig. S5]. **B**) Western blots analysis of phosphorylated-histone H3 (ser10) as a marker of mitotic chromatin condensation. Full blots: [Fig. S5]. Cells were treated 12 h with 40nM SN-38 + 2µM VE-822. Displayed images are cropped from the same blot. β-tubulin serves as a loading control. The β-tubulin-normalized quantifications relative to WT (DMSO) were calculated from 3 independent replicates. **C**) Expression and phosphorylation RPA32 and RPApS4/8 in response to 5 h induction with 40 nM SN-38 ± 2 µM VE-822. Full blots: [Fig. S5]. β-tubulin serves as a loading control. The experiments were repeated 3 times and a representative blot of each protein is shown. Displayed images are cropped from the same blot Full blots: [Fig. S5]. Flow cytometry: co-labelling of RPA32 and RPApS4/8 in WT cells treated with SN-38 + VE-822, same experimental workflow as [Fig. 4C-E].

Figure S5: Annotated, full western blots corresponding to the cropped blots displayed throughout the article.

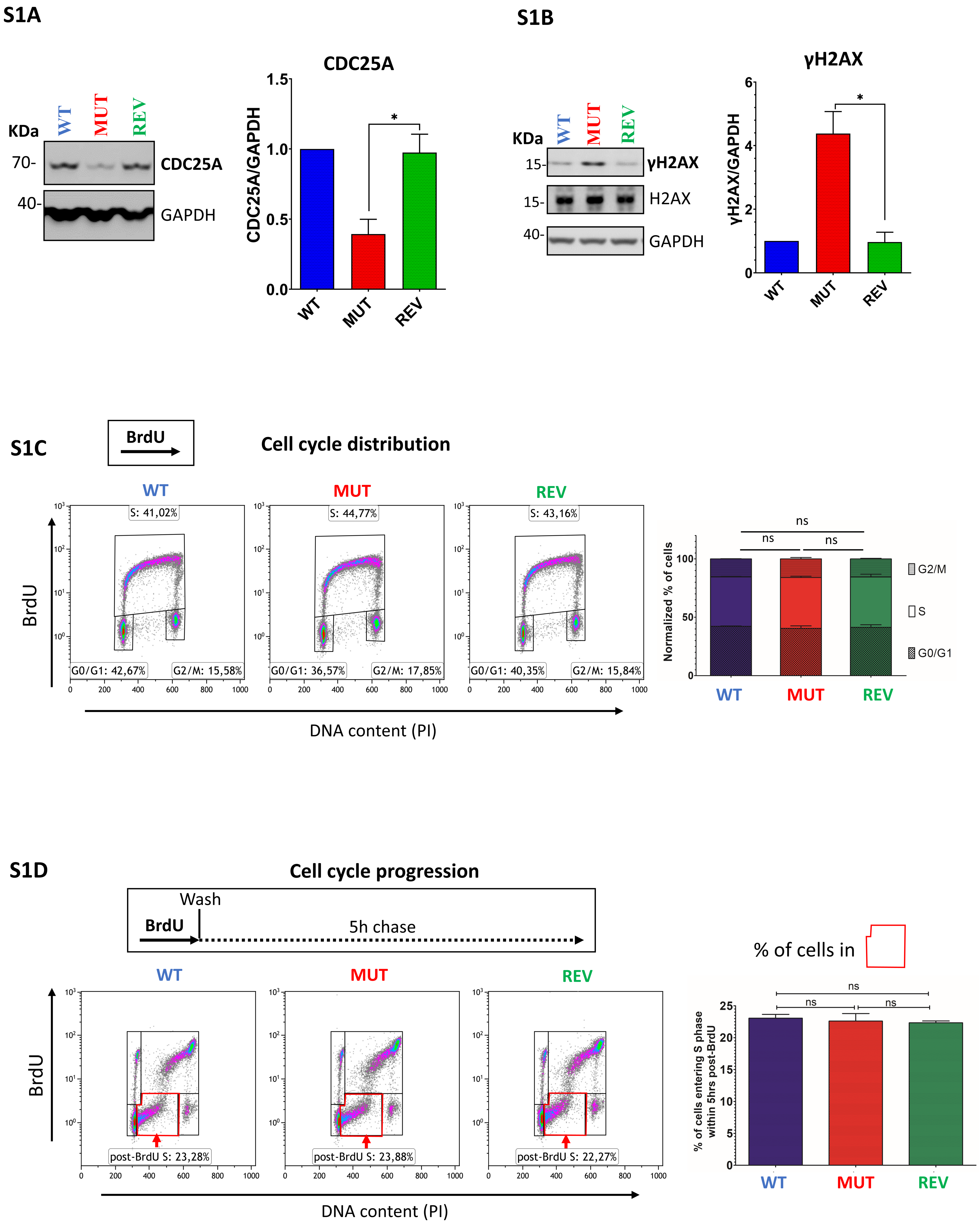
S1A
KDa
WT
MUT
REV
70-
40-
CDC25A
GAPDH
CDC25A
CDC25A/GAPDH
1.5
1.0
0.5
0.0
*
WT
MUT
REV
S1B
KDa
WT
MUT
REV
15-
15-
40-
γH2AX
H2AX
GAPDH
γH2AX
γH2AX/GAPDH
6
4
2
0
*
WT
MUT
REV
S1C
BrdU
Cell cycle distribution
WT
MUT
REV
S: 41,02%
S: 44,77%
S: 43,16%
G0/G1: 42,67%
G2/M: 15,58%
G0/G1: 36,57%
G2/M: 17,85%
G0/G1: 40,35%
G2/M: 15,84%
BrdU
DNA content (PI)
Normalized % of cells
100
50
0
ns
ns
ns
G2/M
S
G0/G1
WT
MUT
REV
S1D
Cell cycle progression
Wash
BrdU
5h chase
WT
MUT
REV
post-BrdU S: 23,28%
post-BrdU S: 23,88%
post-BrdU S: 22,27%
BrdU
DNA content (PI)
% of cells in
% of cells entering S phase within 5hrs post-BrdU
25
20
15
10
5
0
ns
ns
ns
WT
MUT
REV

Figure S1

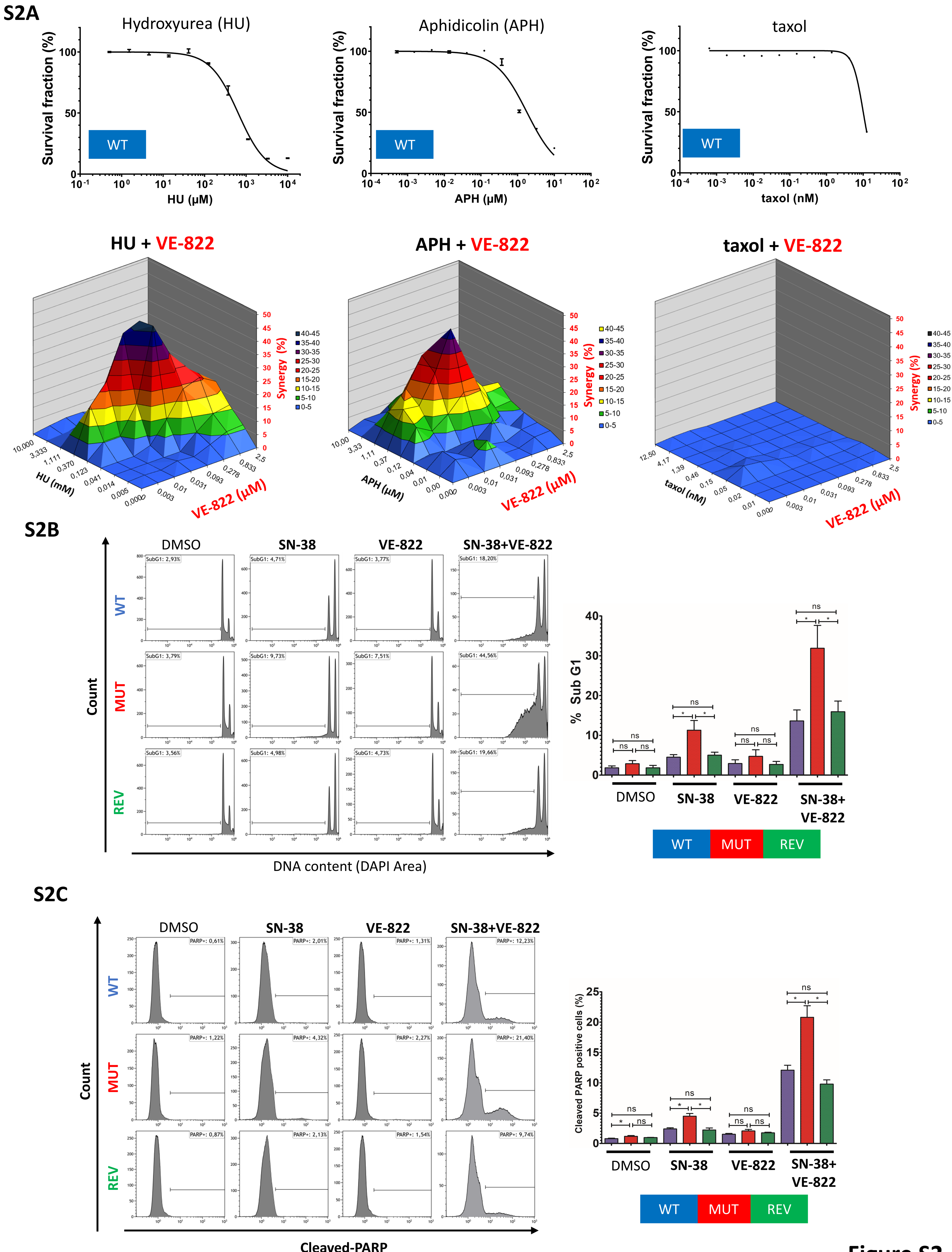
S2A
Hydroxyurea (HU)
Aphidicolin (APH)
taxol
Survival fraction (%)
WT
HU (µM)
APH (µM)
taxol (nM)
HU + VE-822
APH + VE-822
taxol + VE-822
Synergy (%)
HU (mM)
APH (µM)
taxol (nM)
VE-822 (µM)
S2B
DMSO
SN-38
VE-822
SN-38+VE-822
WT
MUT
REV
Count
DNA content (DAPI Area)
% Sub G1
S2C
Cleaved-PARP
Cleaved PARP positive cells (%)

Figure S2

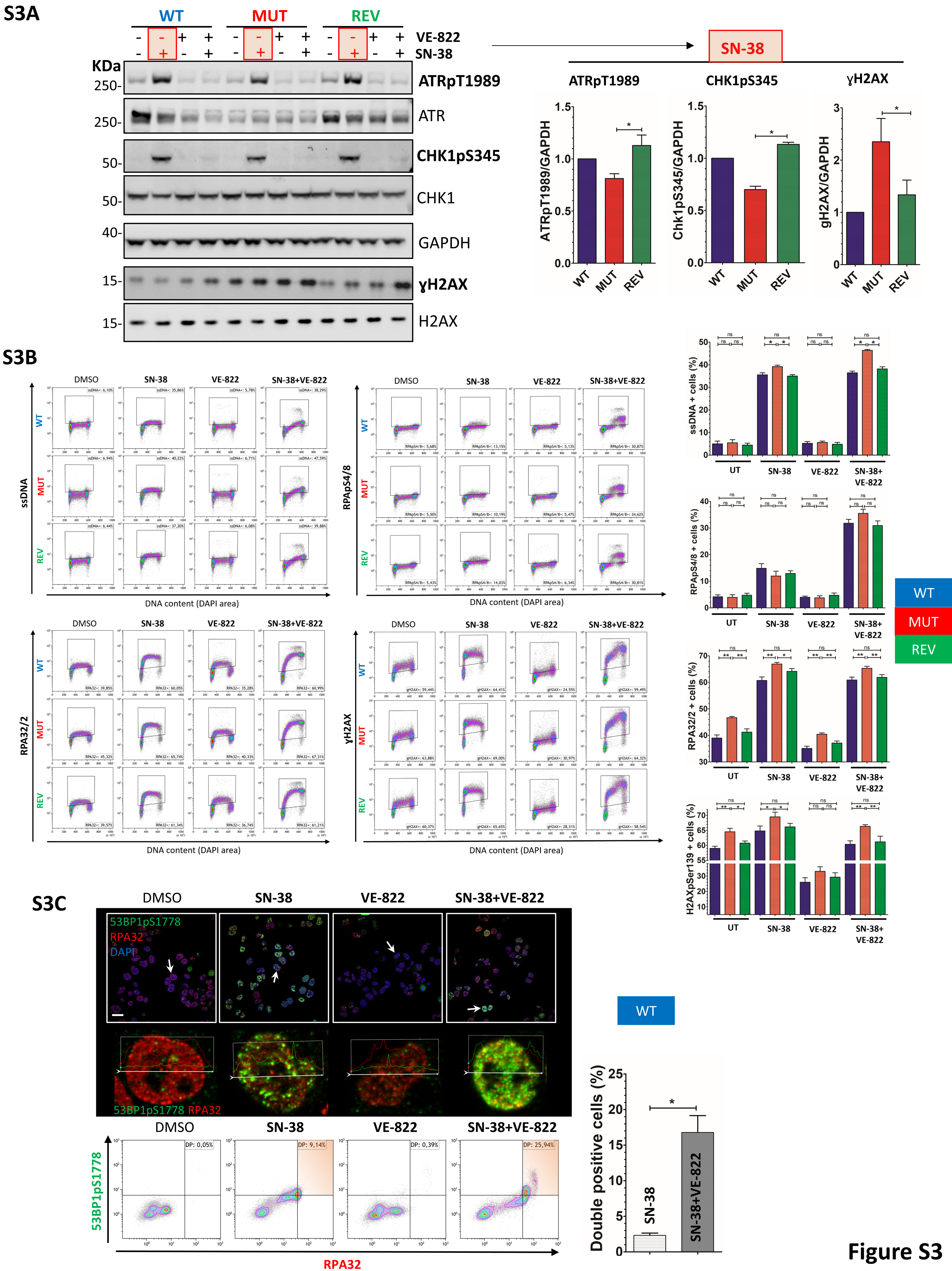
S3A
WT
MUT
REV
VE-822
SN-38
KDa
250-
50-
40-
15-
ATRpT1989
ATR
CHK1pS345
CHK1
GAPDH
γH2AX
H2AX
SN-38
ATRpT1989/GAPDH
Chk1pS345/GAPDH
gH2AX/GAPDH
S3B
DMSO
SN-38
VE-822
SN-38+VE-822
ssDNA
RPApS4/8
RPA32/2
γH2AX
DNA content (DAPI area)
ssDNA + cells (%)
RPApS4/8 + cells (%)
RPA32/2 + cells (%)
H2AXpSer139 + cells (%)
UT
SN-38+
VE-822
ns
S3C
53BP1pS1778
RPA32
DAPI
53BP1pS1778 RPA32
DP: 0,05%
DP: 9,14%
DP: 0,39%
DP: 25,94%
Double positive cells (%)
SN-38+VE-822

Figure S3

S4A

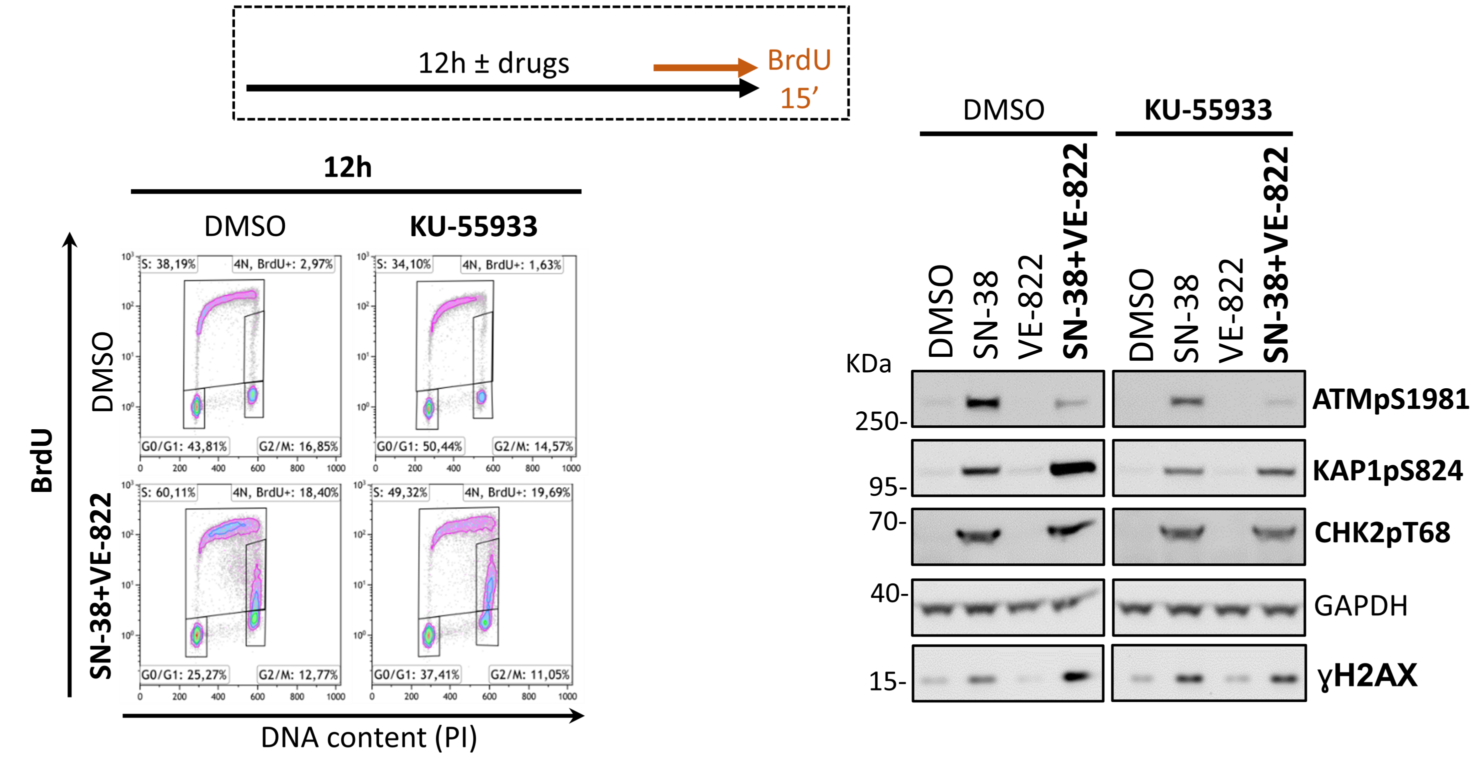

S4B

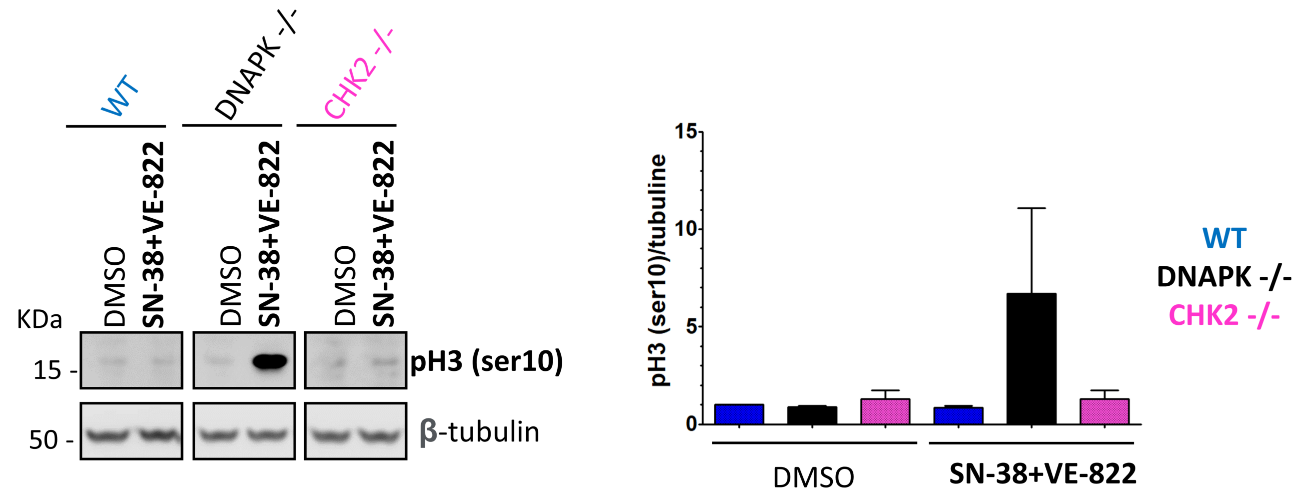

S4C

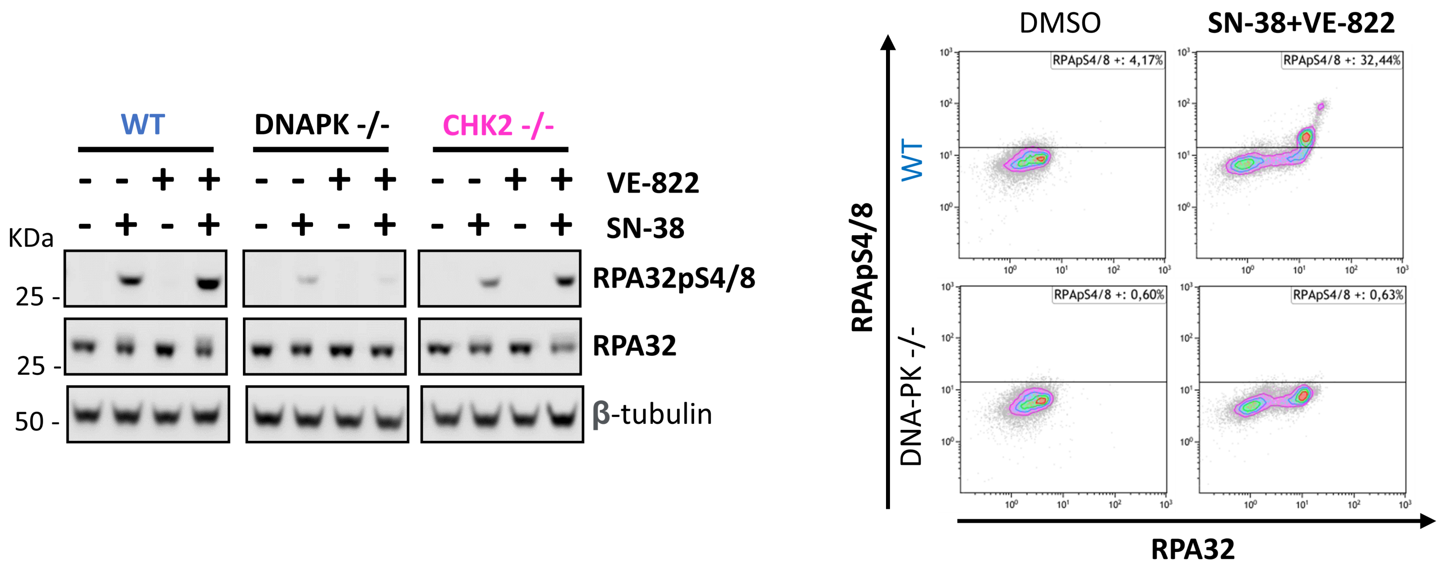

Figure S4

Figure S5: full blots (1)

Blots in [Fig. 1C]

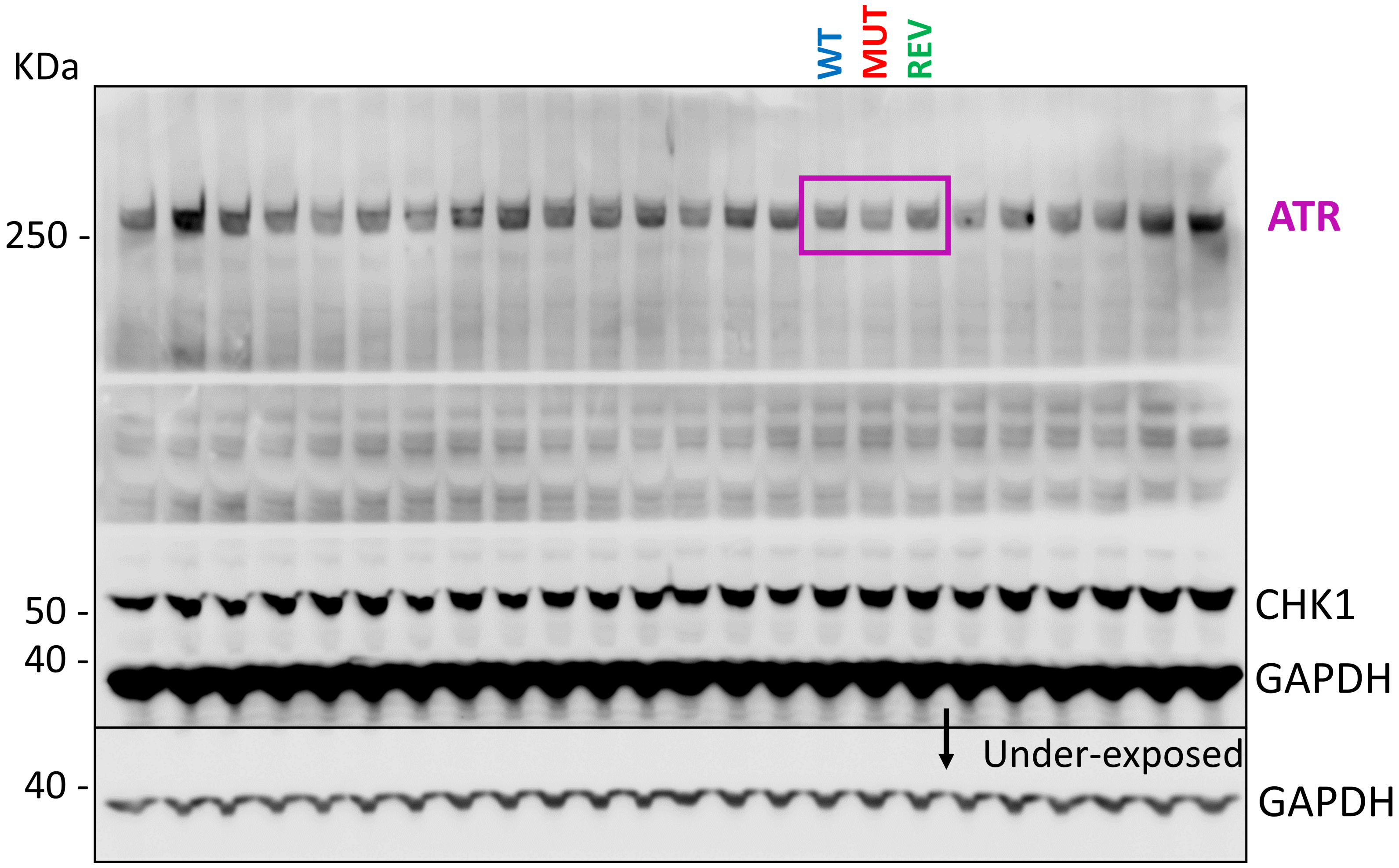

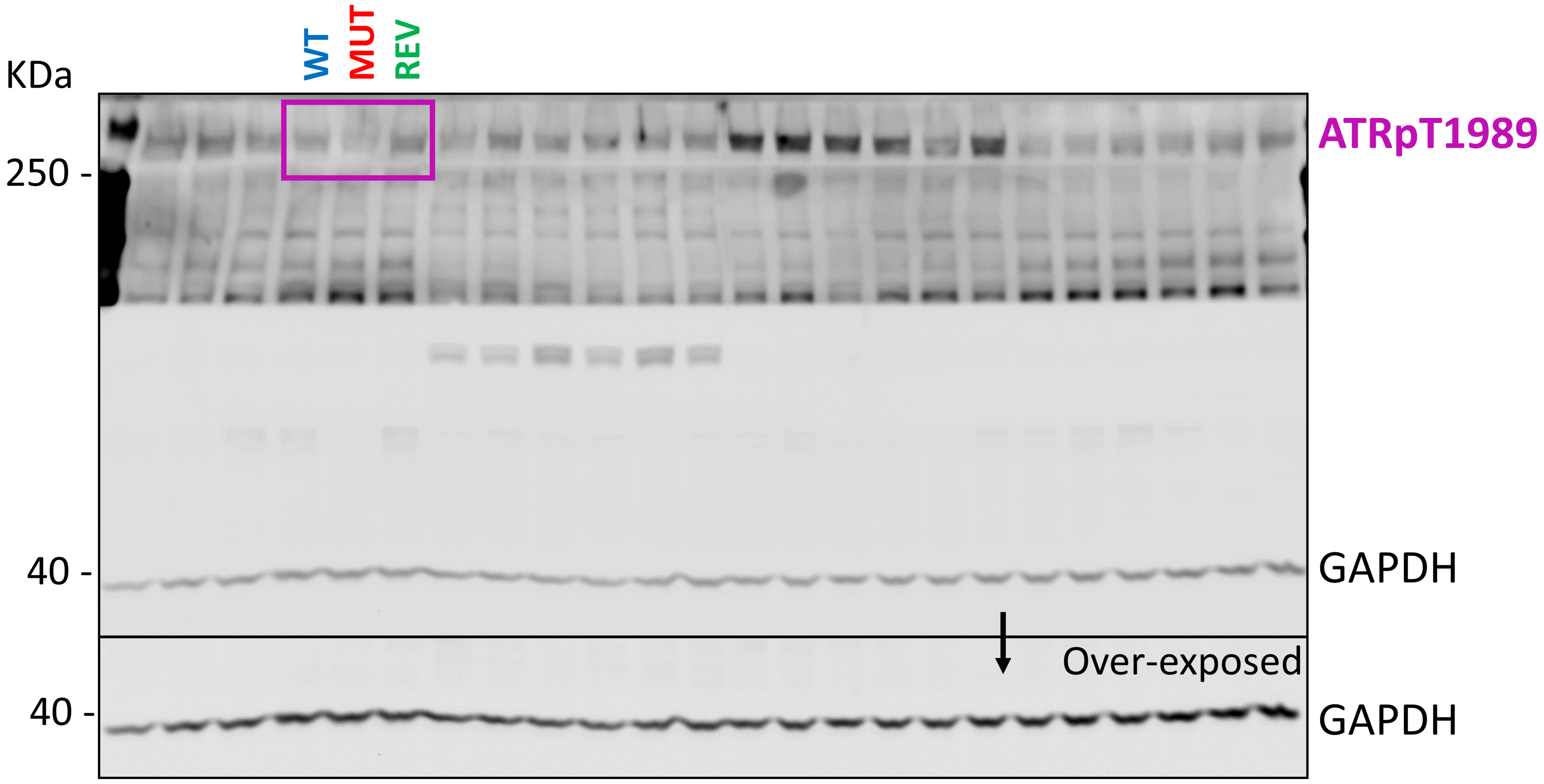

Figure S5: full blots (2)

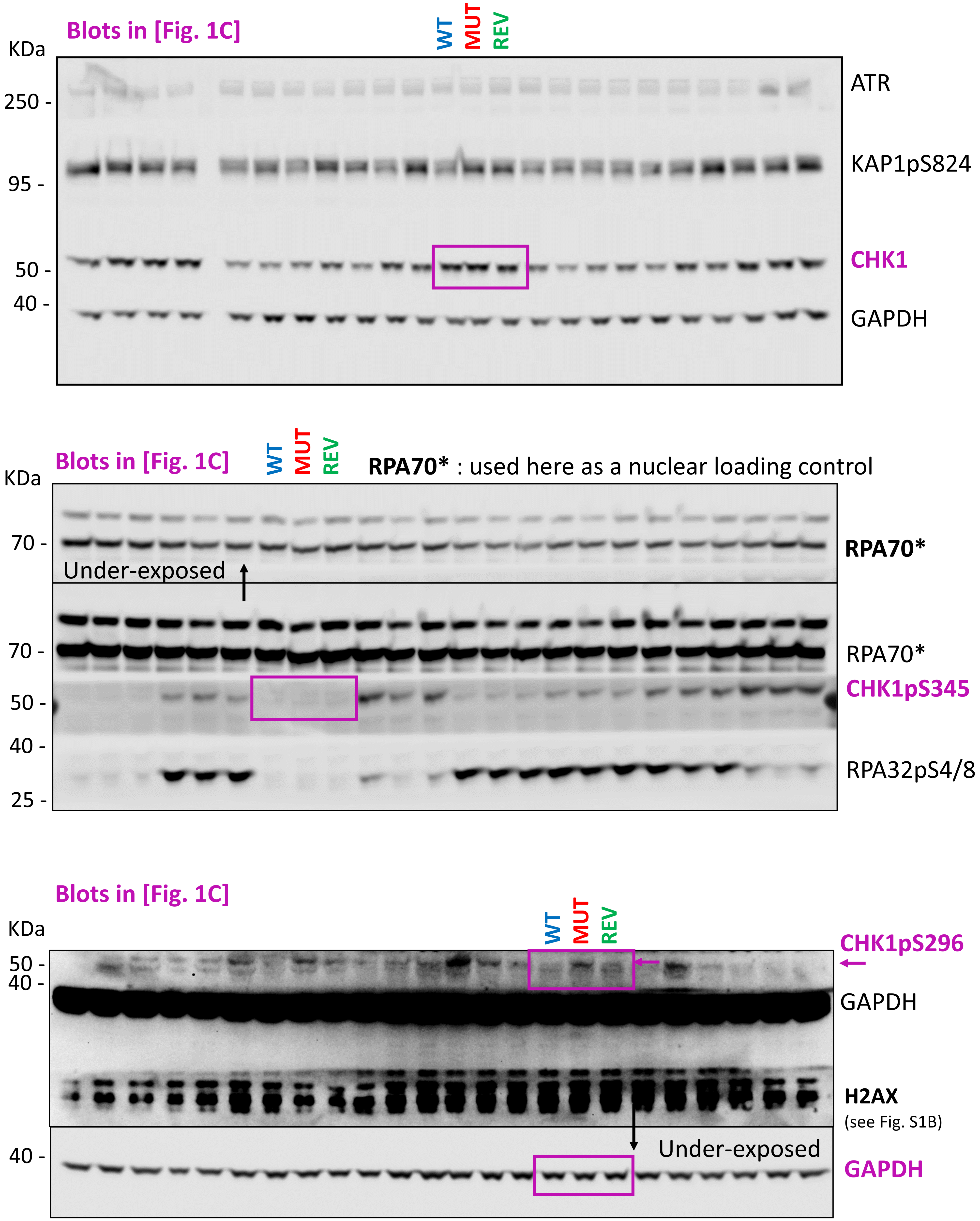

Figure S5: full blots (3)

Blots in [Fig. 1C]

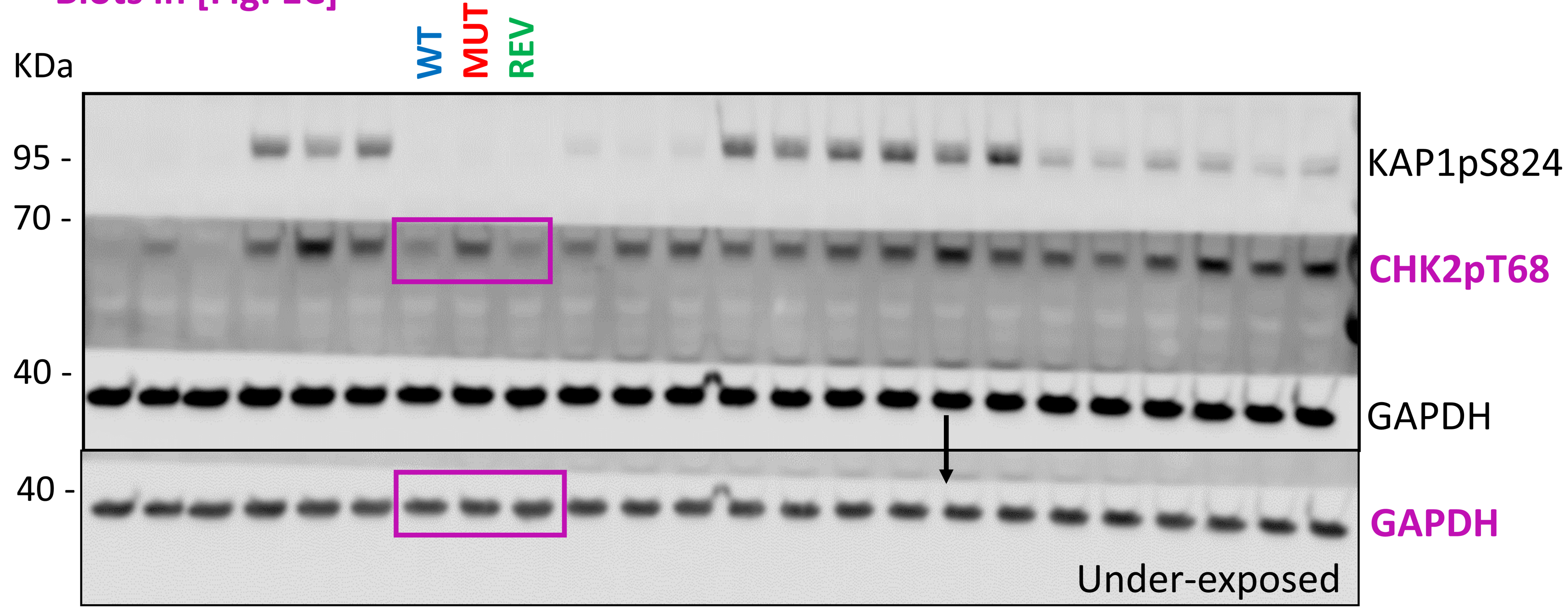

Blots in [Fig. 1C]

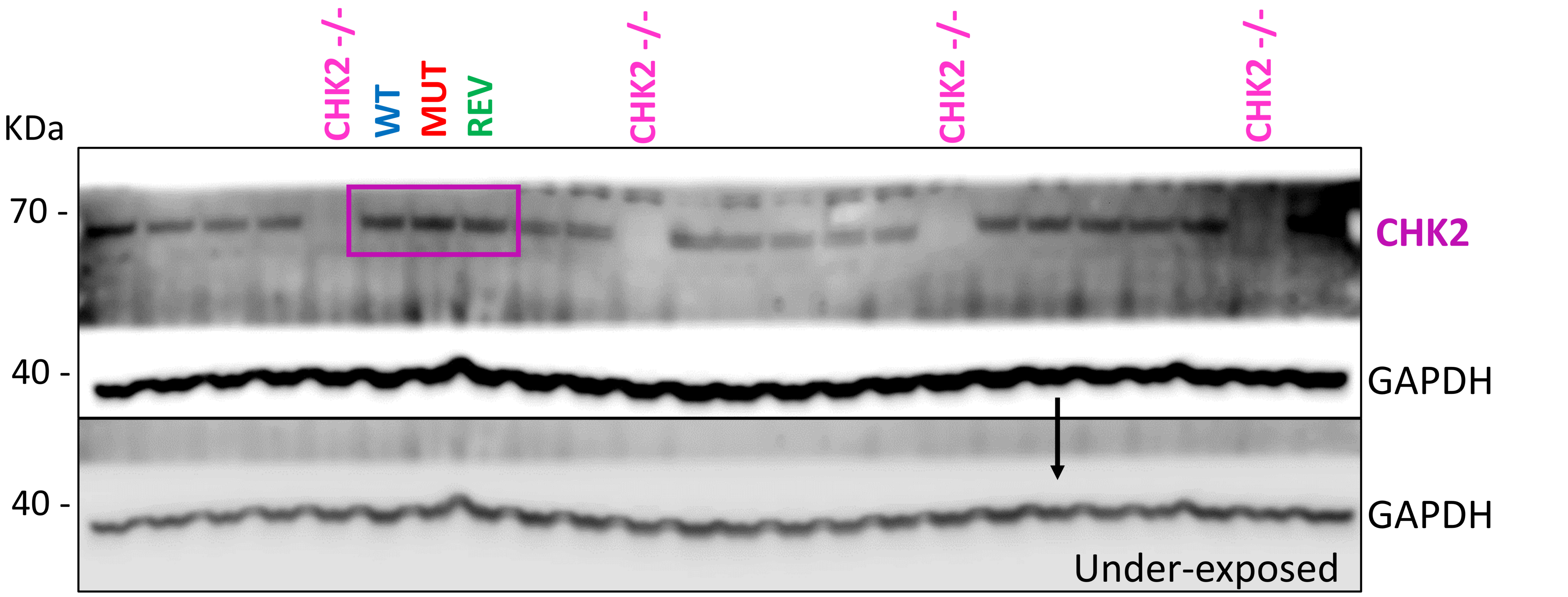

Blots in [Fig. 1C]

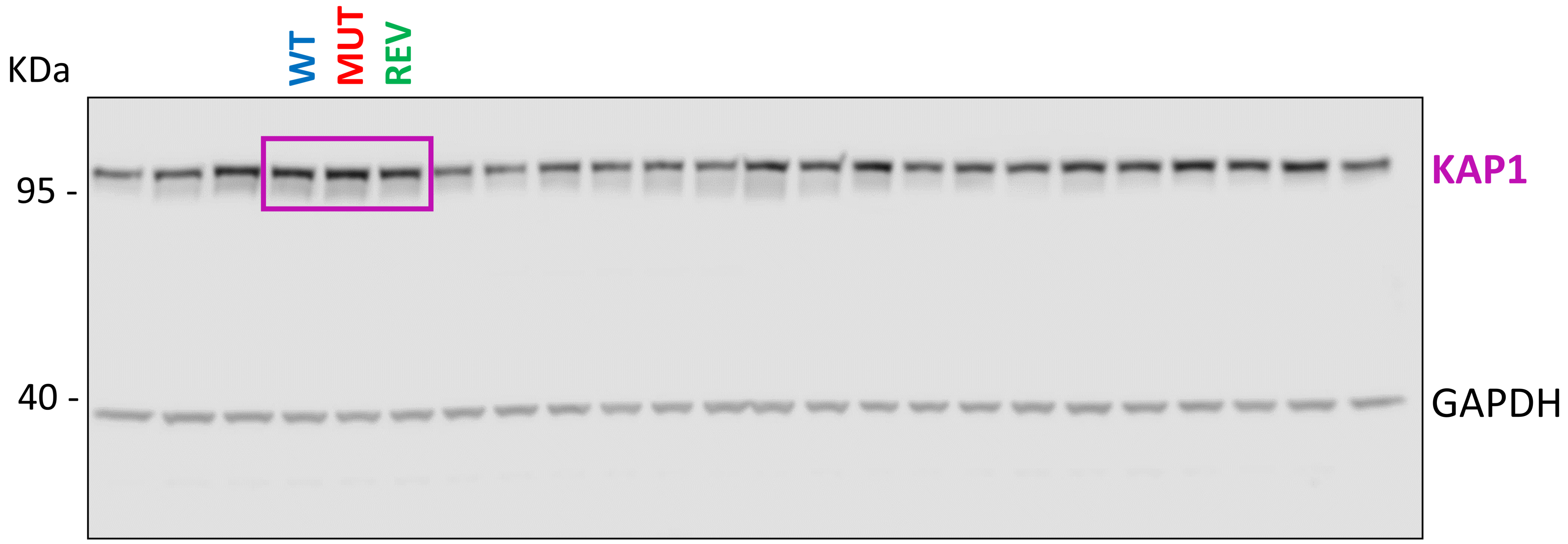

Figure S5: full blots (4)

Blots in [Fig. 1C]

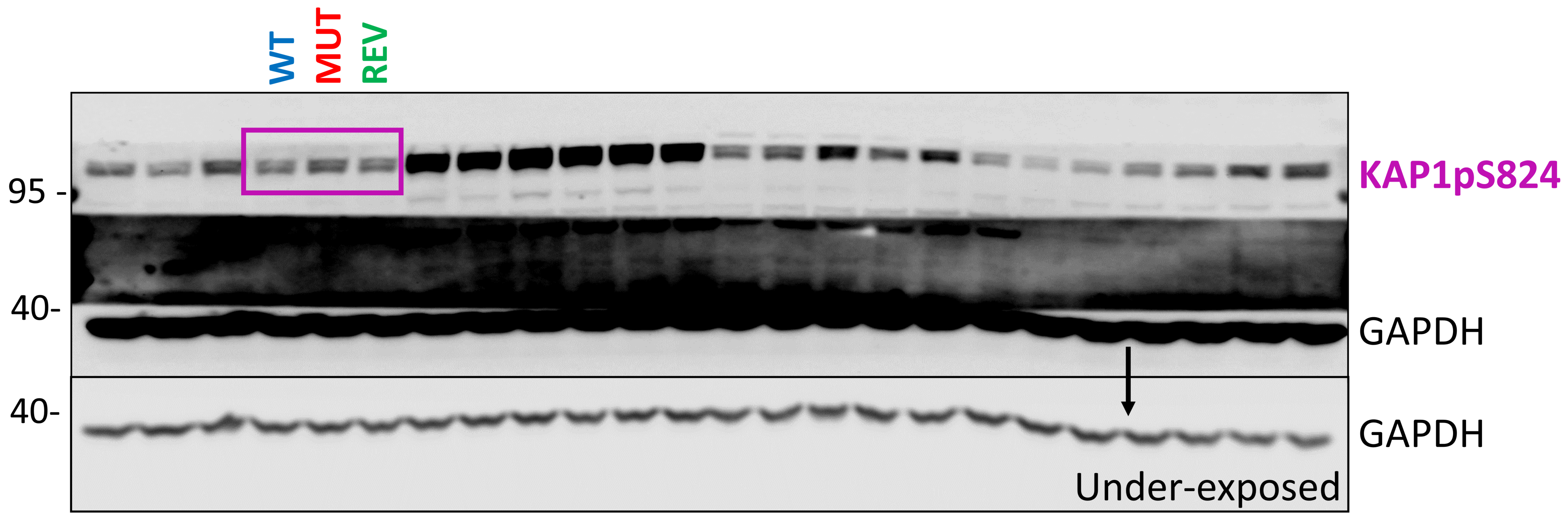

Blots in [Fig. 1C]

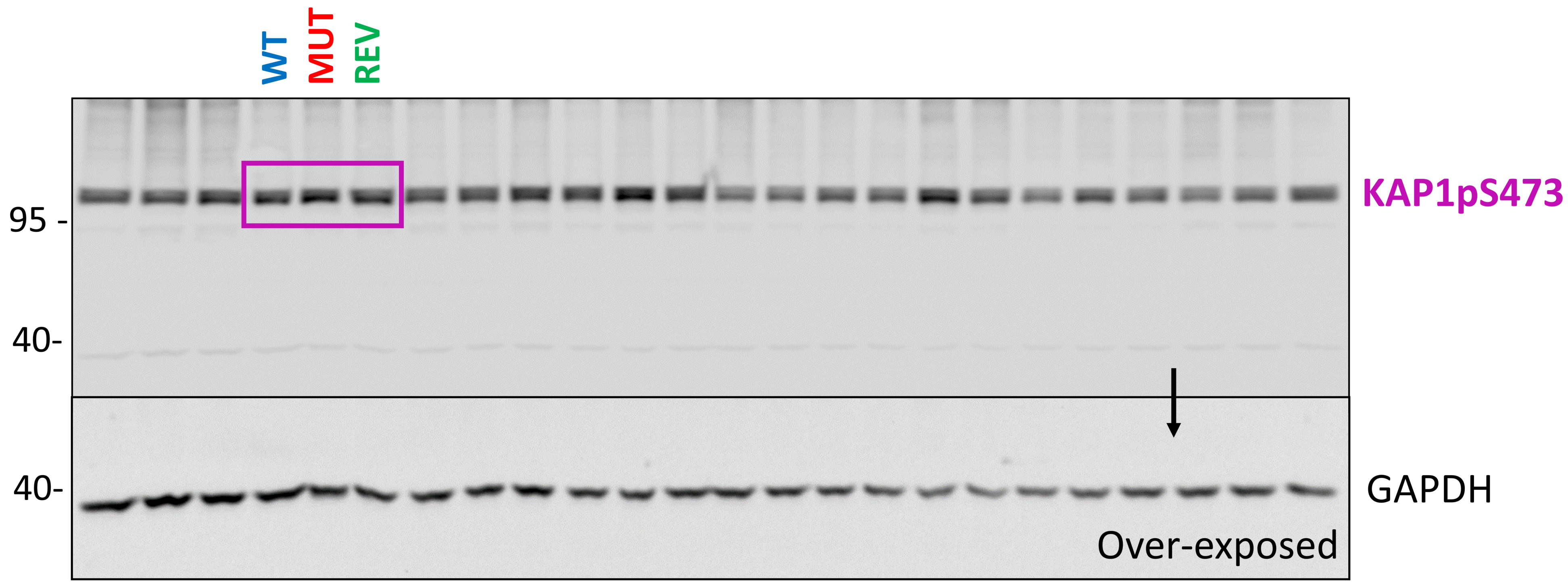

Figure S5: full blots (5)

Blots in [Fig. 3B]

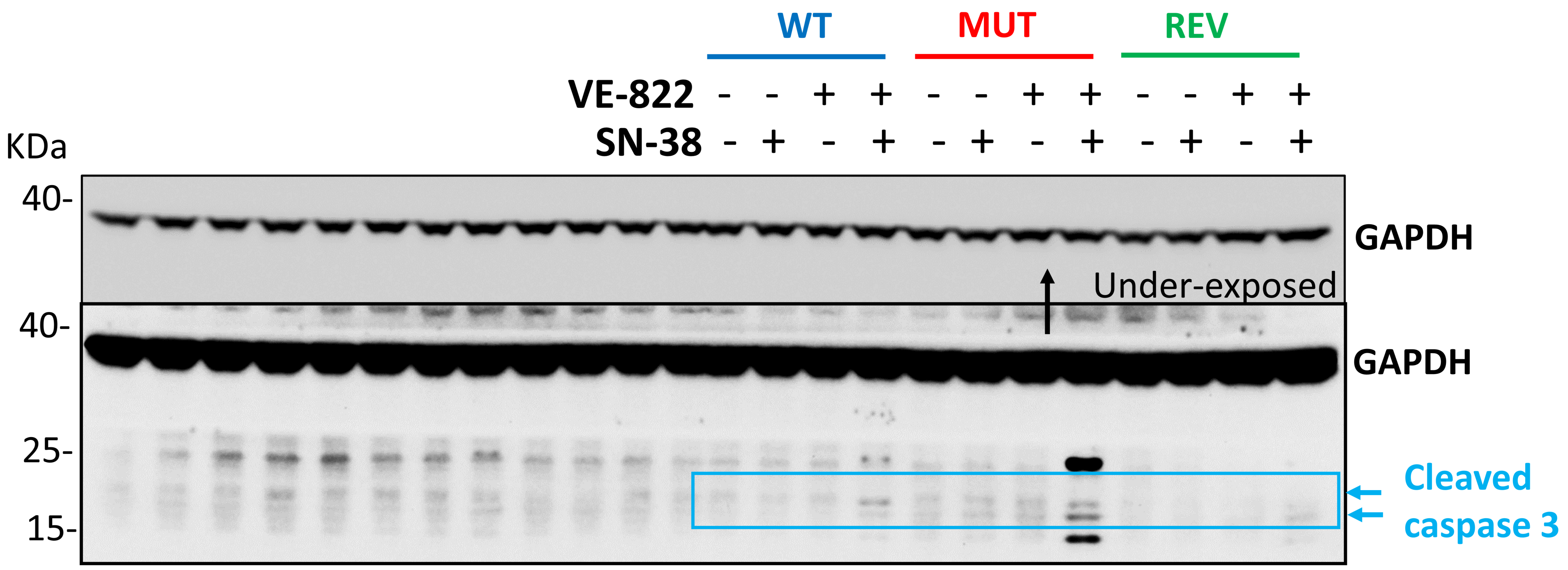

Blots in [Fig. 3B]

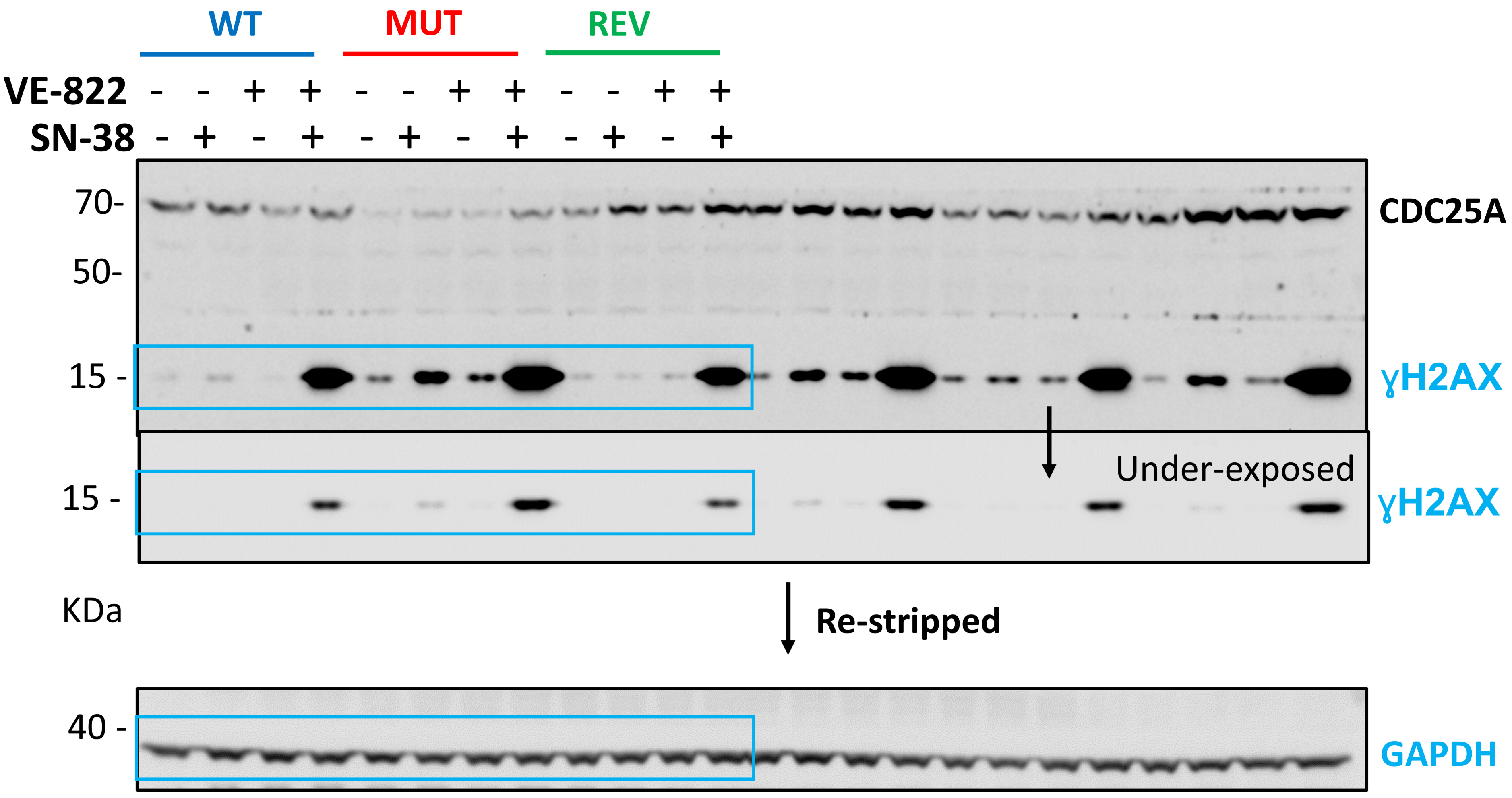

Figure S5: full blots (6)

Blots in [Fig. 3E]

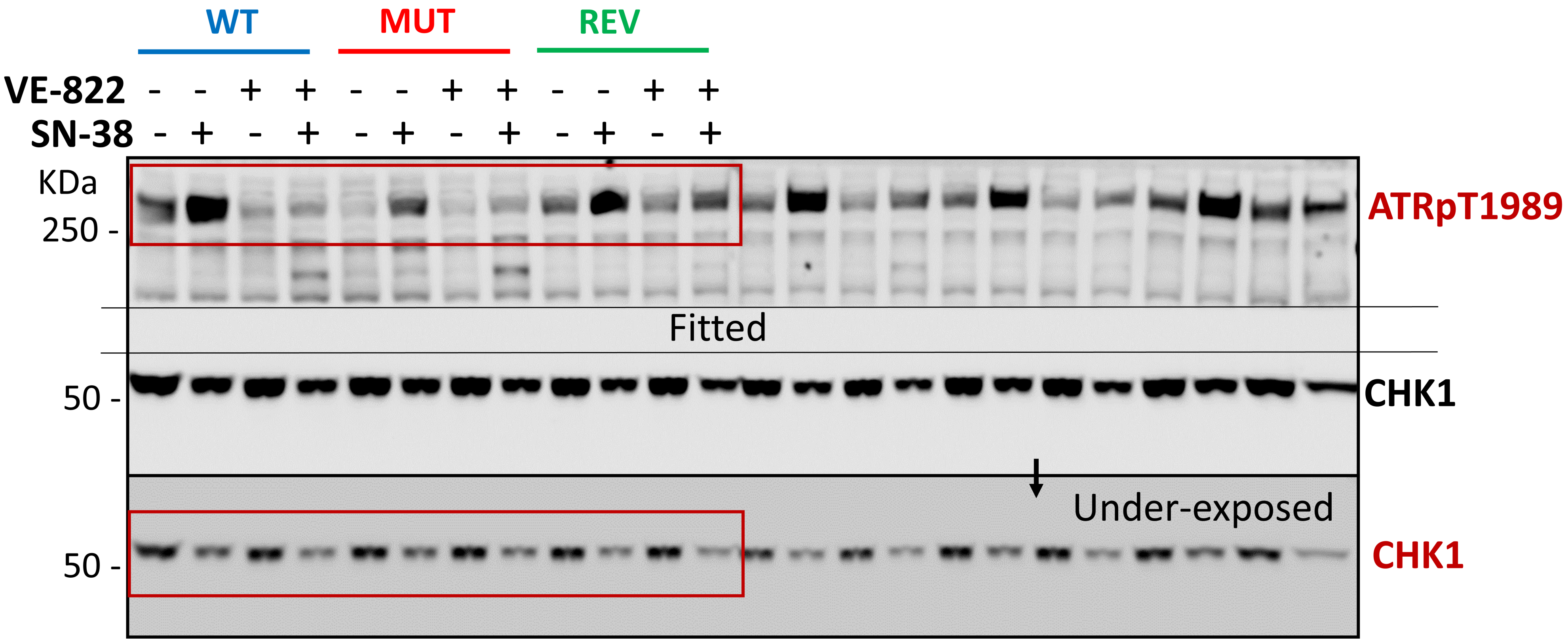

Blots in [Fig. 3E]

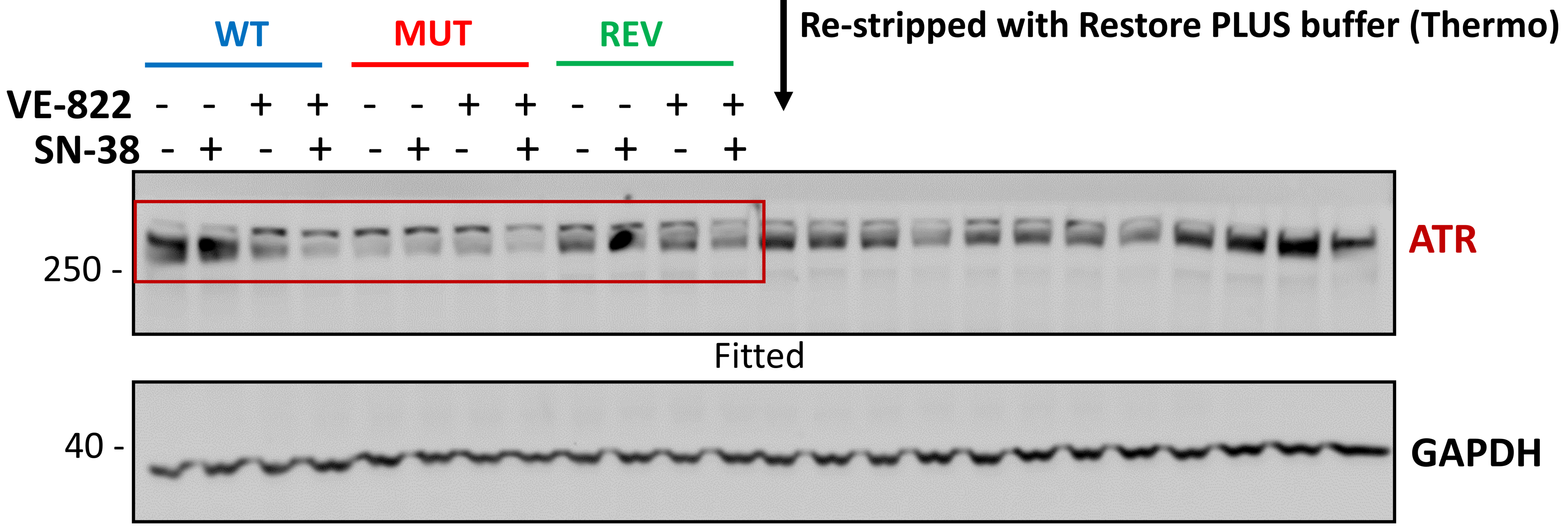

Blots in [Fig. 3E]

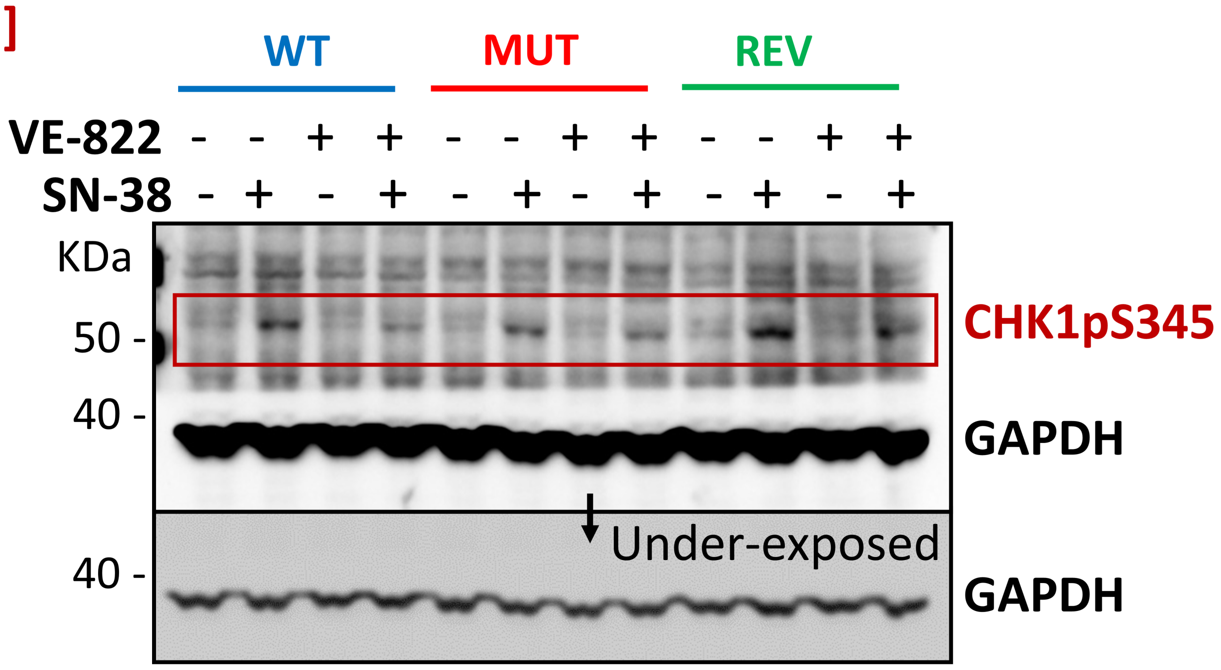

Figure S5: full blots (7)

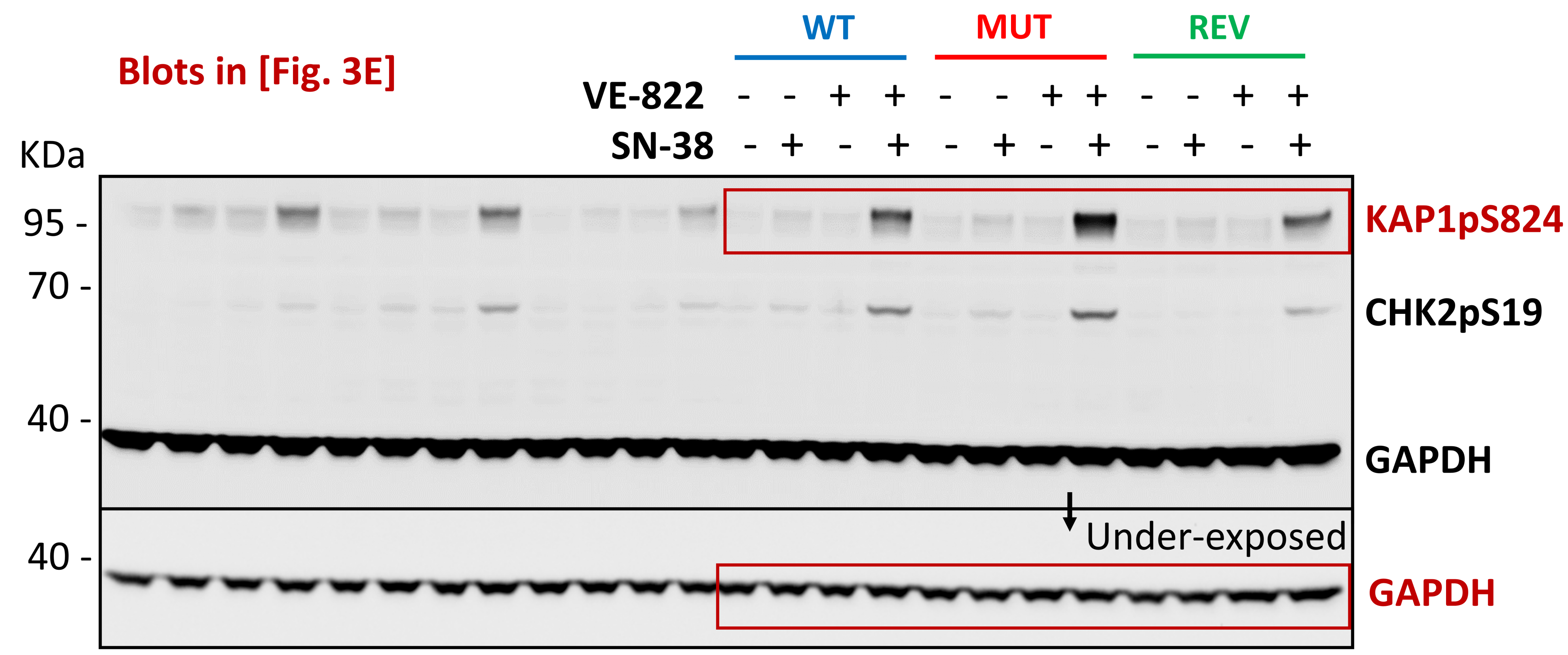

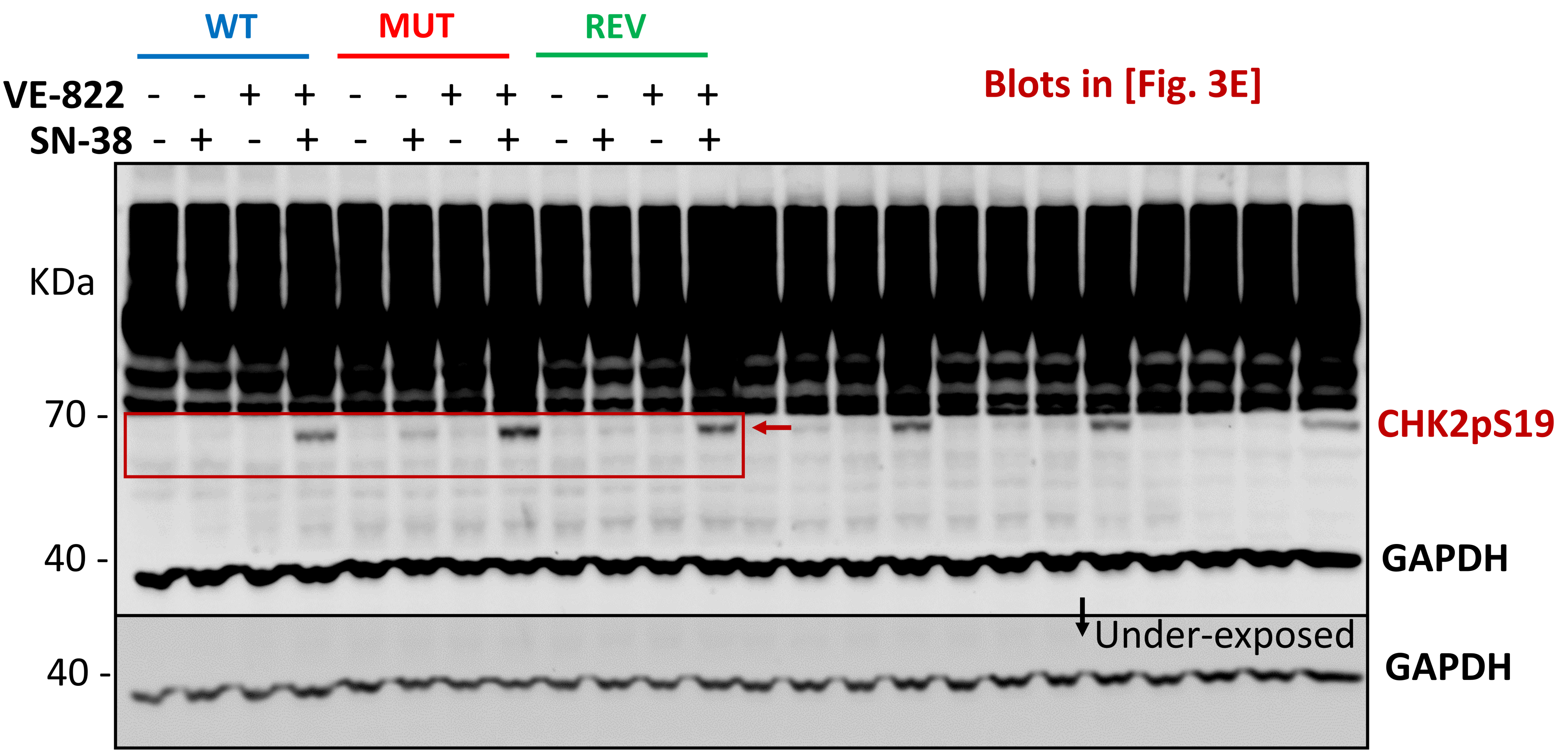

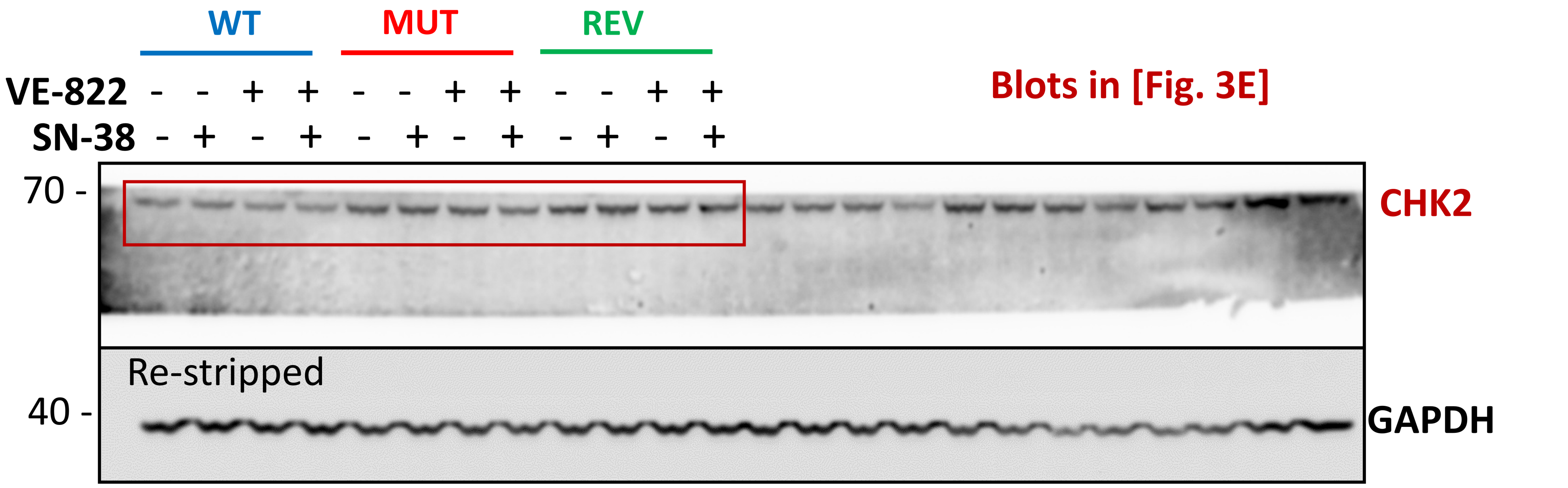

Figure S5: full blots (8)

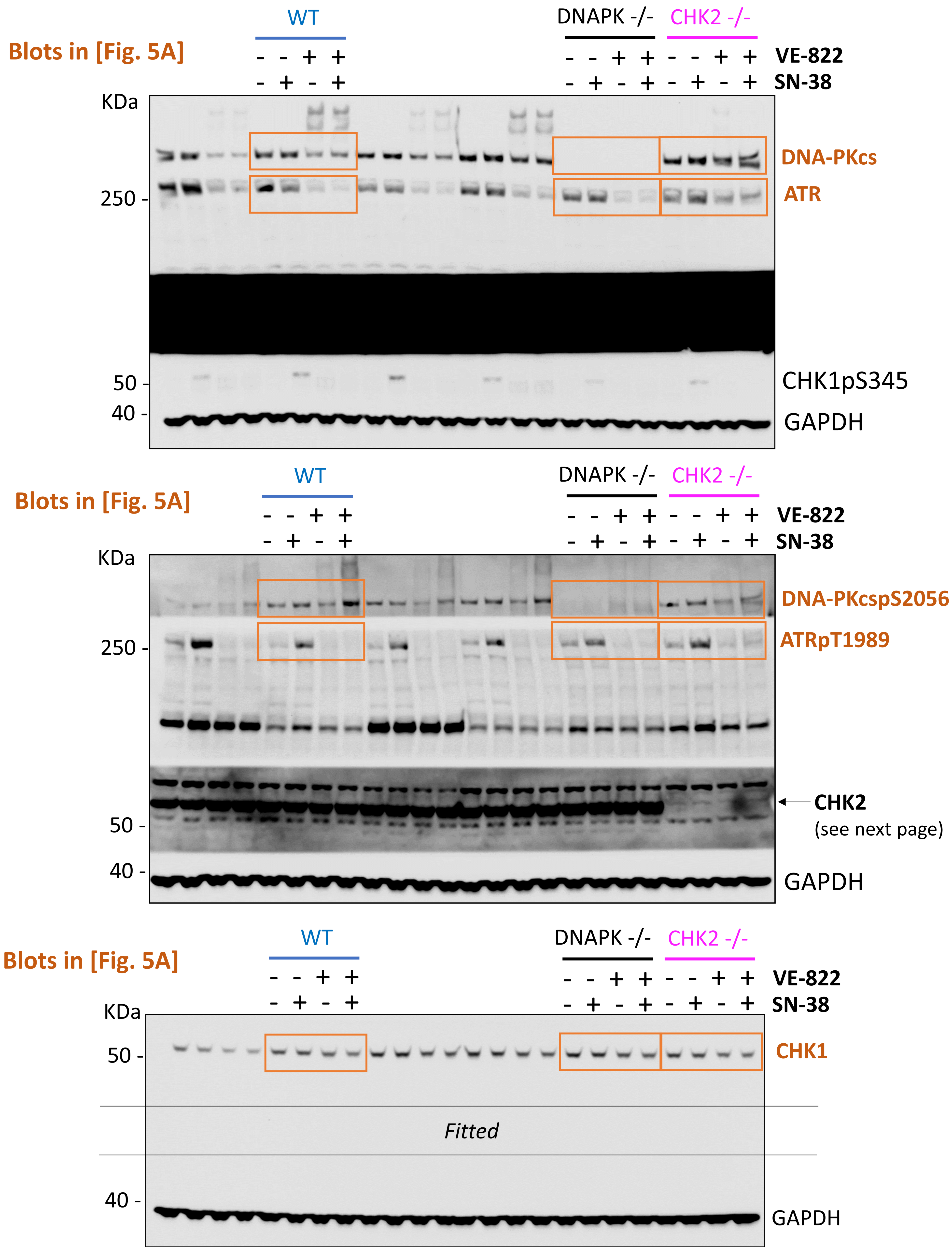

Figure S5: full blots (9)

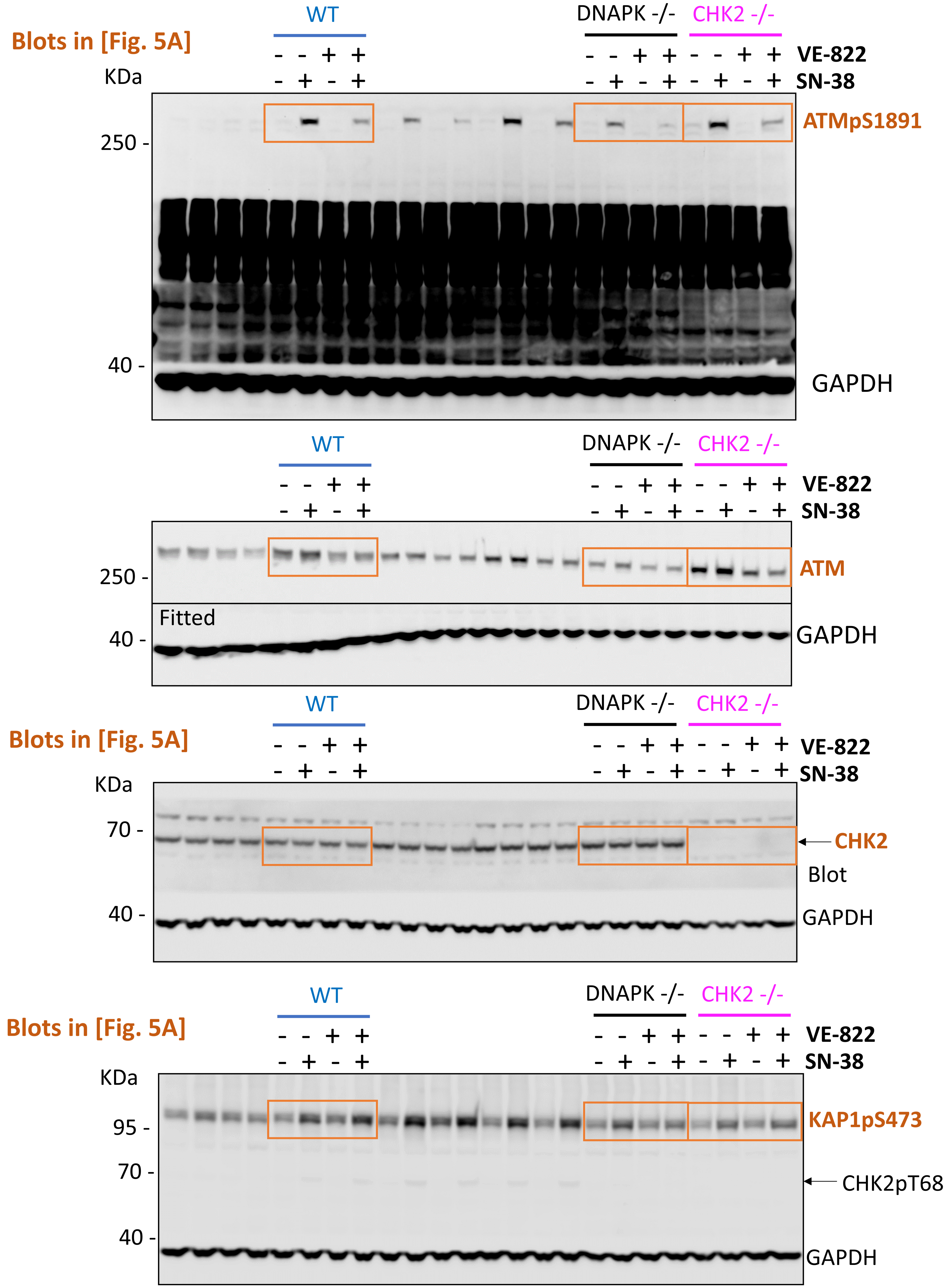

Figure S5: full blots (10)

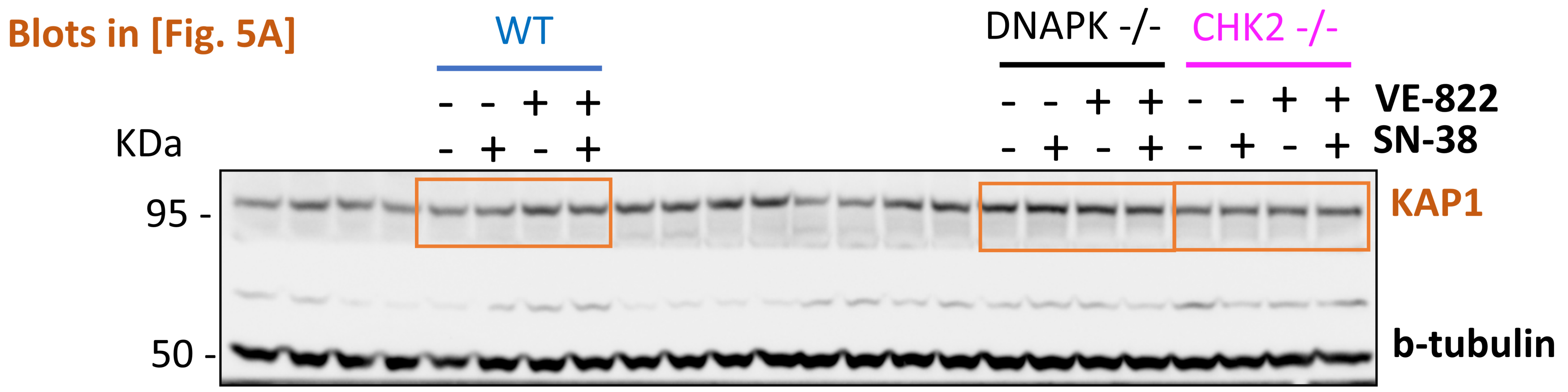

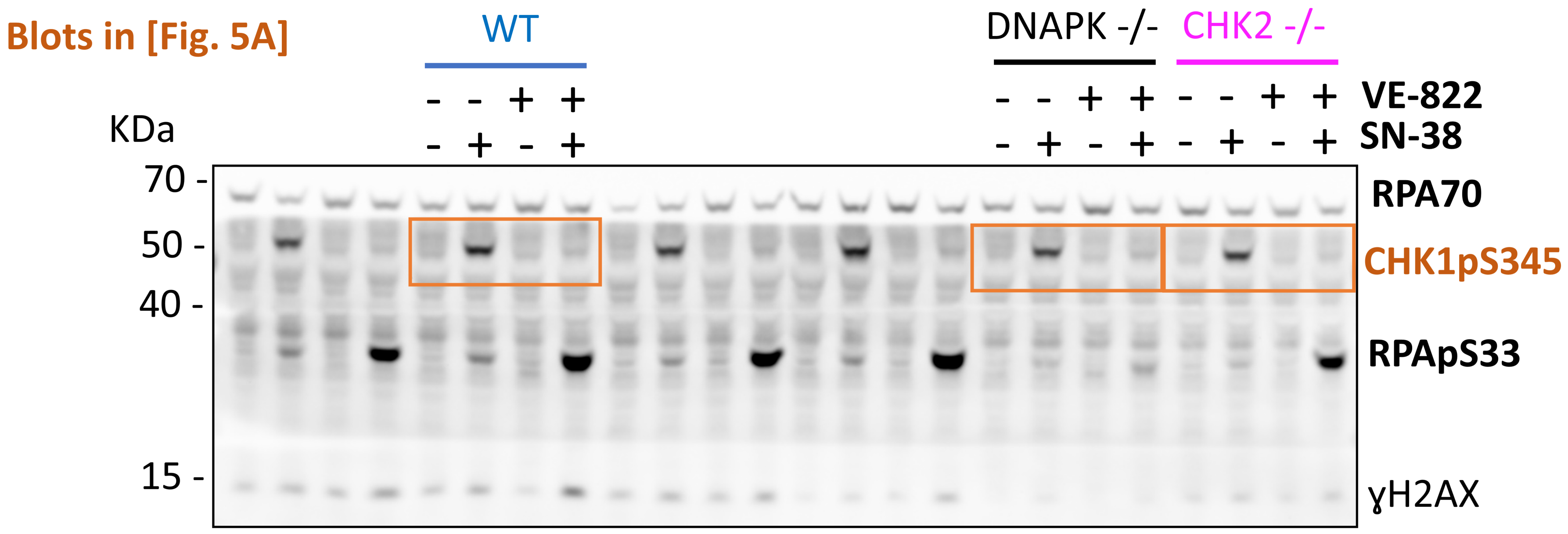

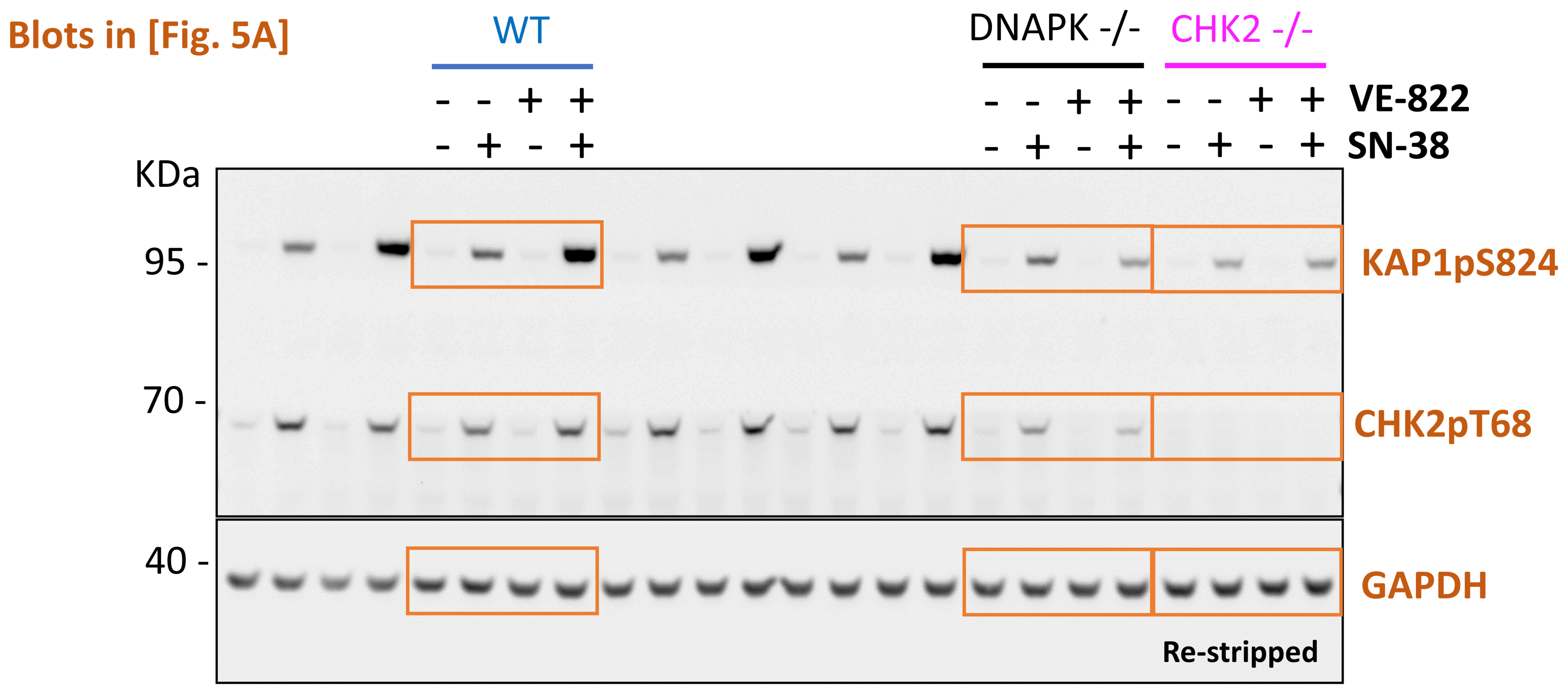

Figure S5: full blots (11)

Blots in [Fig. S1A]

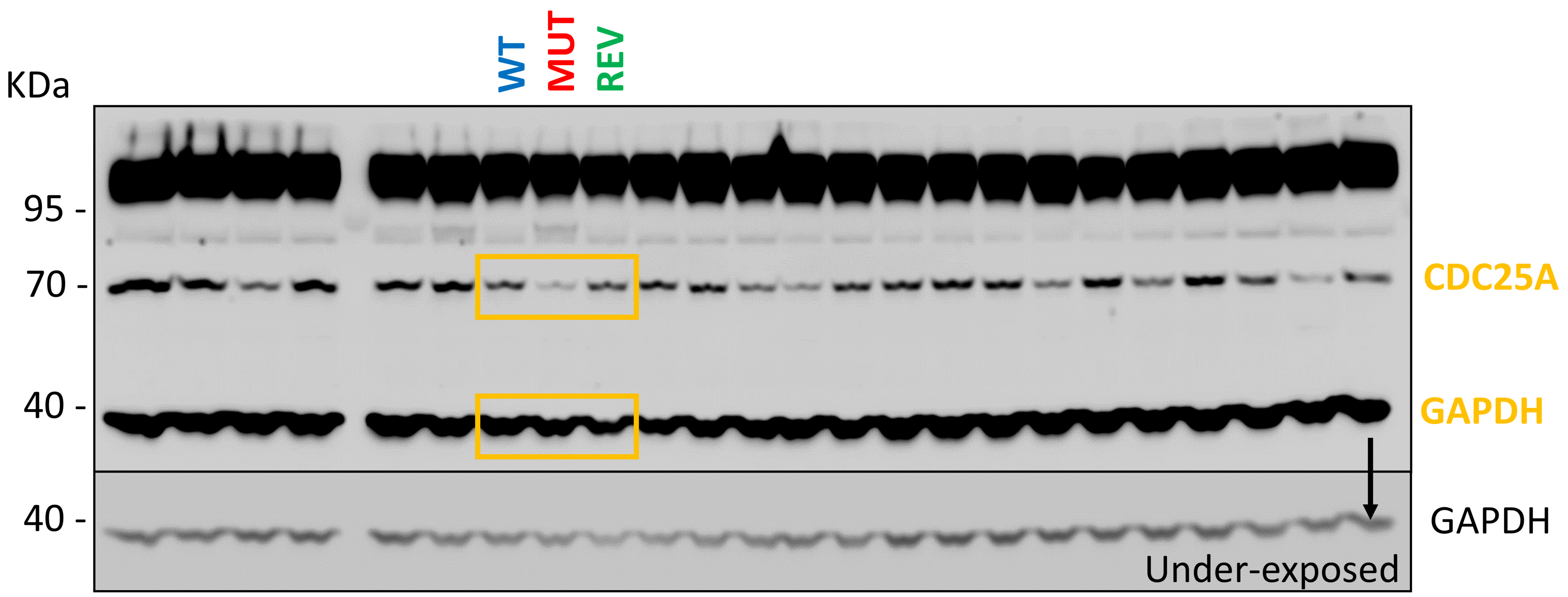

Blots in [Fig. S1B]

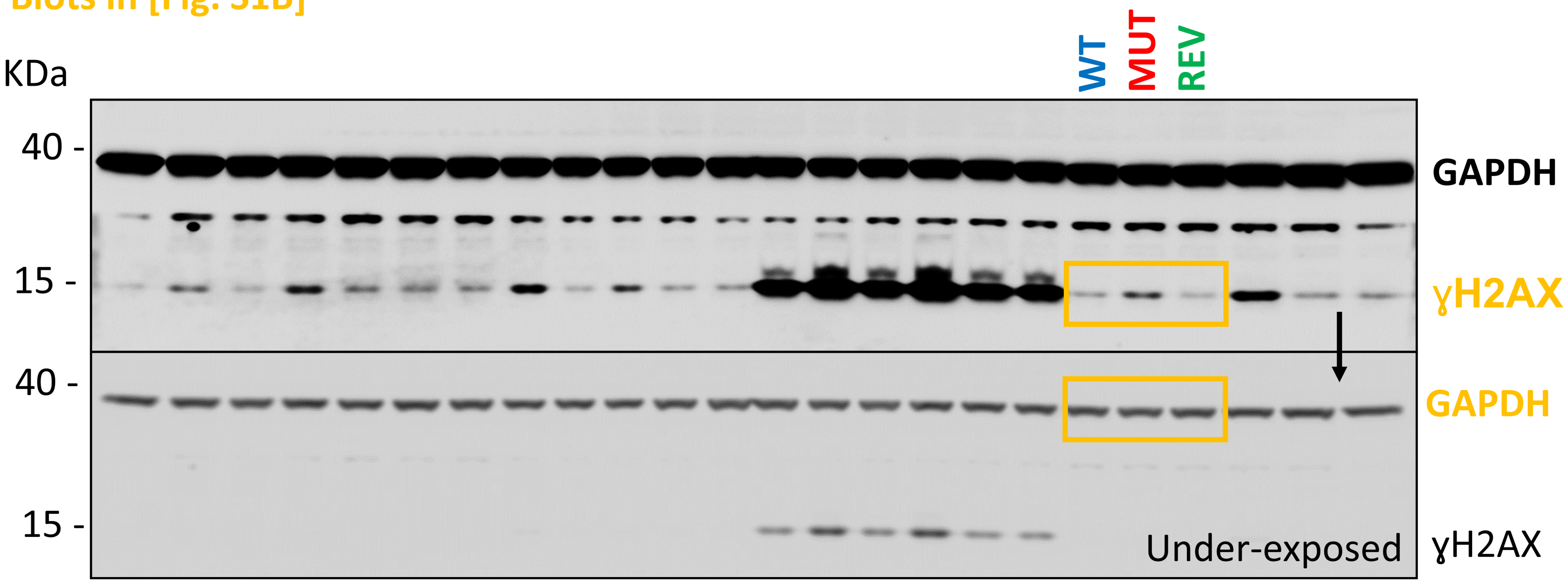

Blots in [Fig. S1B]

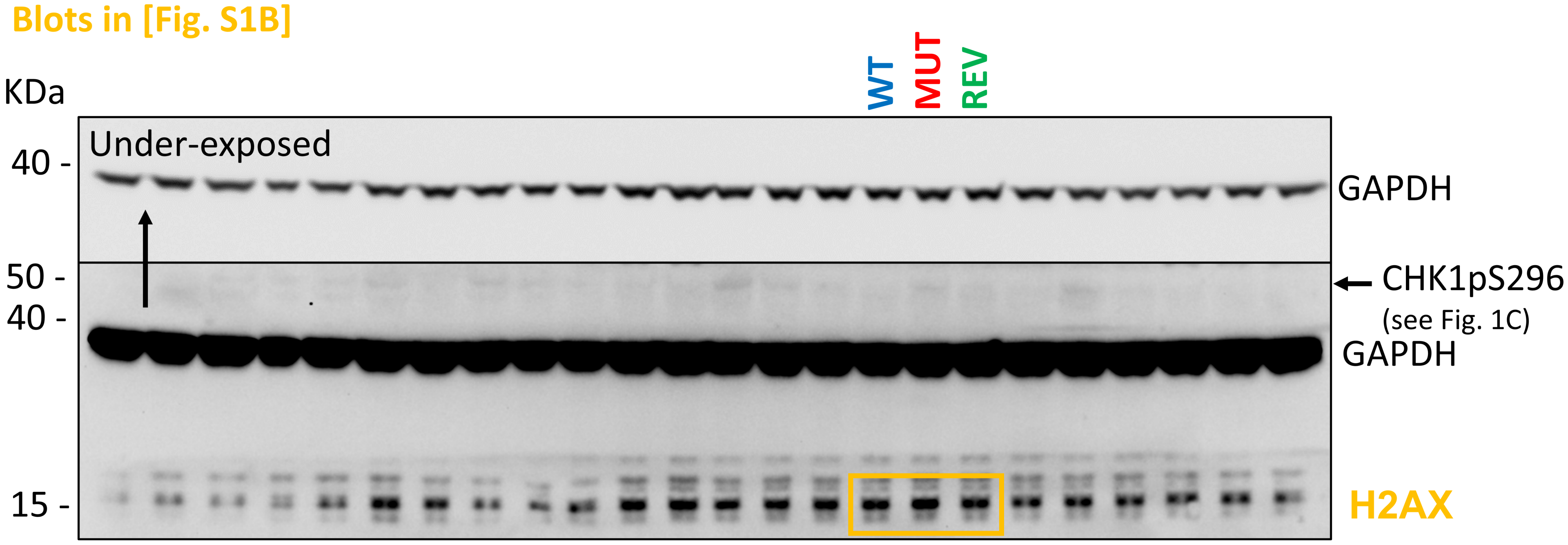

Figure S5: full blots (12)

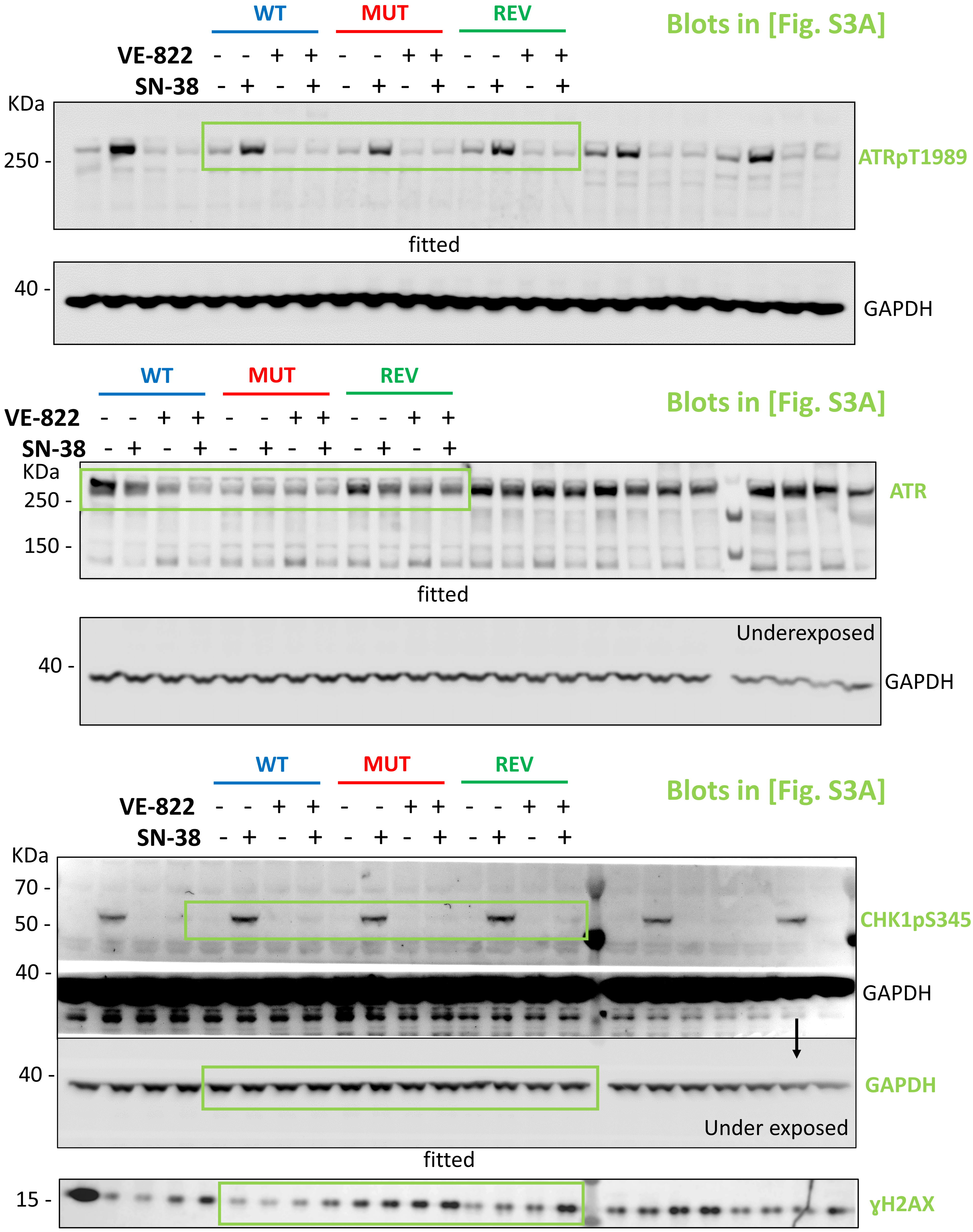

Figure S5: full blots (13)

Blots in [Fig. S3A]

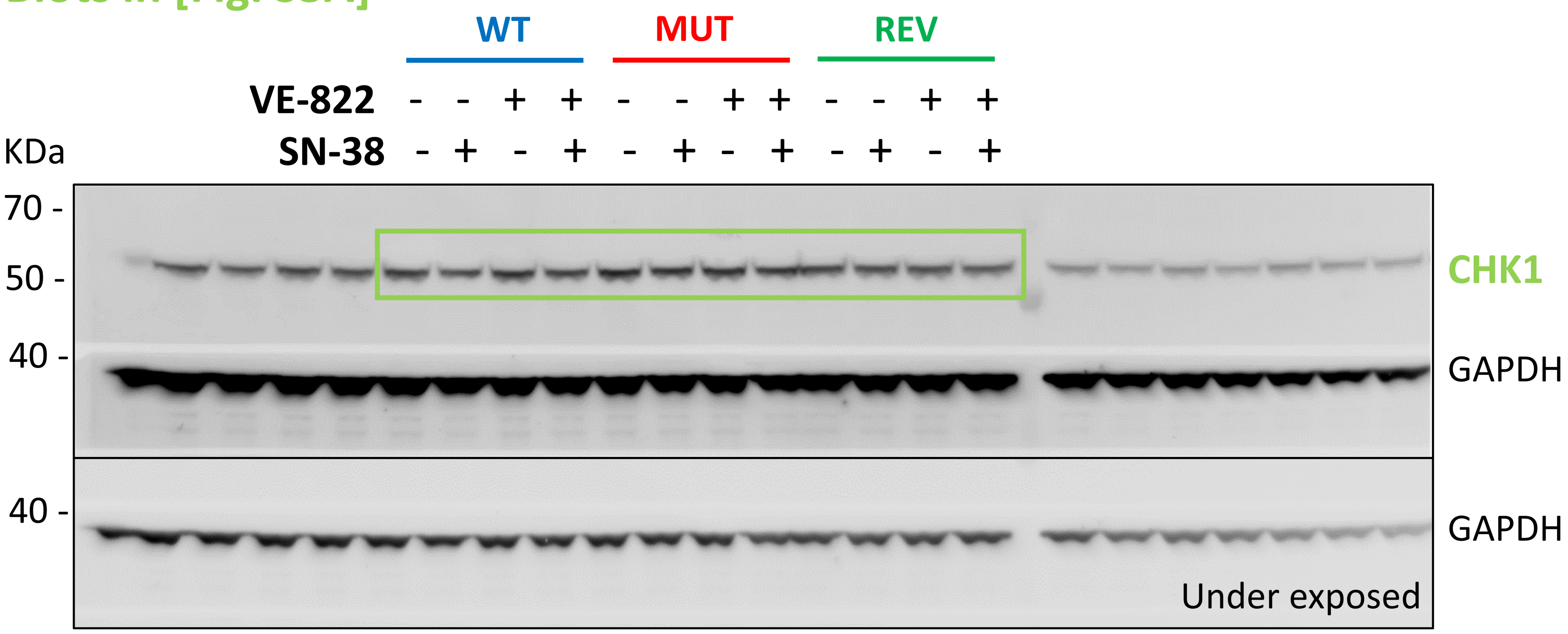

Blots in [Fig. S3A]

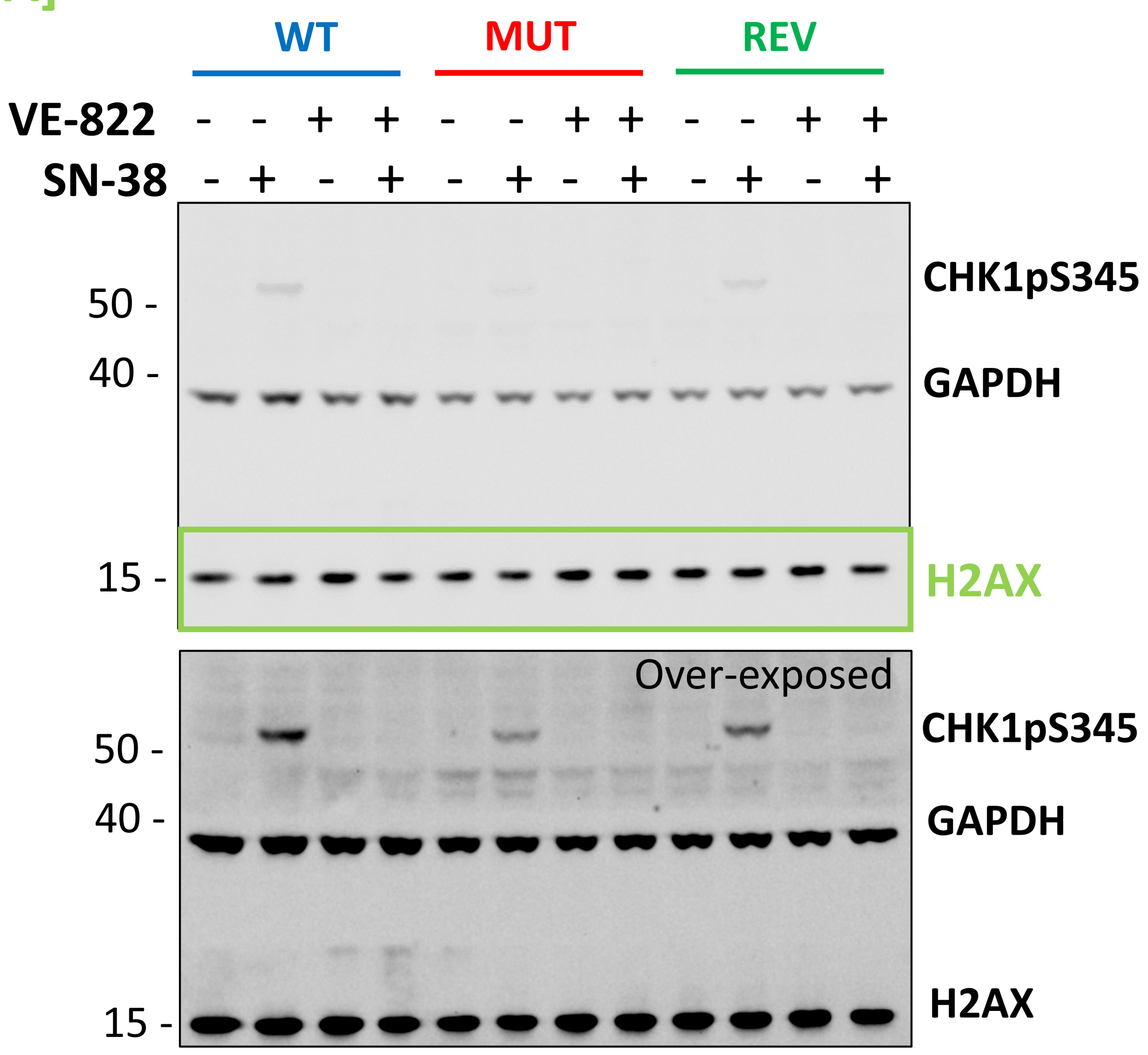

Figure S5: full blots (14)

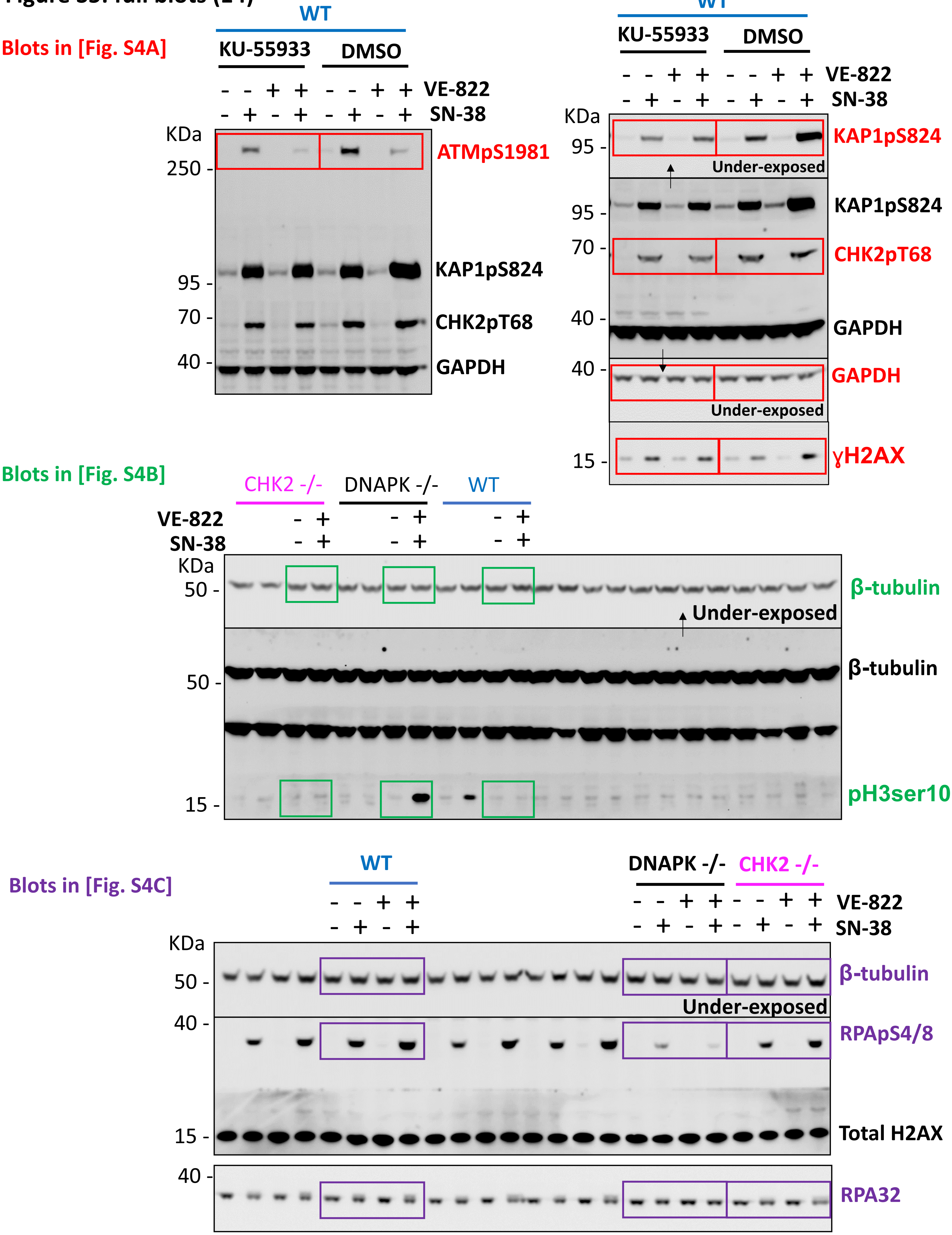

Supplement: Supplementary file 1 — Supplementary Information. [file 41598_2022_9308_MOESM1_ESM.pdf]
